# Movements of Ancient Human Endogenous Retroviruses Detected in SOX2-Expressing Cells

**DOI:** 10.1128/jvi.00356-22

**Published:** 2022-04-14

**Authors:** Kazuaki Monde, Yorifumi Satou, Mizuki Goto, Yoshikazu Uchiyama, Jumpei Ito, Taku Kaitsuka, Hiromi Terasawa, Nami Monde, Shinya Yamaga, Tomoya Matsusako, Fan-Yan Wei, Ituro Inoue, Kazuhito Tomizawa, Akira Ono, Takumi Era, Tomohiro Sawa, Yosuke Maeda

**Affiliations:** a Department of Microbiology, Faculty of Life Sciences, Kumamoto University, Kumamoto, Japan; b Joint Research Center for Human Retrovirus Infection, Kumamoto University, Kumamoto, Japan; c Department of Medical Image Sciences, Kumamoto University, Kumamoto, Japan; d Department of Molecular Physiology, Faculty of Life Sciences, Kumamoto University, Kumamoto, Japan; e Department of Cell Modulation, Institute of Molecular Embryology and Genetics (IMEG), Kumamoto University, Kumamoto, Japan; f Division of Human Genetics, National Institute of Genetics, Shizuoka, Japan; g Division of Systems Virology, Department of Infectious Disease Control, International Research Center for Infectious Diseases, Institute of Medical Science, The University of Tokyo, Tokyo, Japan; h School of Pharmacy at Fukuoka, International University of Health and Welfare, Okawa, Fukuoka, Japan; i Department of Microbiology and Immunology, University of Michigan Medical School, Ann Arbor, Michigan, USA; Ulm University Medical Center

**Keywords:** HERV, LTR, SOX2, iPSC, retrotransposon, teratocarcinoma

## Abstract

Human endogenous retroviruses (HERVs) occupy approximately 8% of the human genome. HERVs, transcribed in early embryos, are epigenetically silenced in somatic cells, except under pathological conditions. HERV-K is thought to protect embryos from exogenous viral infection. However, uncontrolled HERV-K expression in somatic cells has been implicated in several diseases. Here, we show that SOX2, which plays a key role in maintaining the pluripotency of stem cells, is critical for HERV-K LTR5Hs. HERV-K undergoes retrotransposition within producer cells in the absence of Env expression. Furthermore, we identified new HERV-K integration sites in long-term culture of induced pluripotent stem cells that express SOX2. These results suggest that the strict dependence of HERV-K on SOX2 has allowed HERV-K to protect early embryos during evolution while limiting the potentially harmful effects of HERV-K retrotransposition on host genome integrity in these early embryos.

**IMPORTANCE** Human endogenous retroviruses (HERVs) account for approximately 8% of the human genome; however, the physiological role of HERV-K remains unknown. This study found that HERV-K LTR5Hs and LTR5B were transactivated by SOX2, which is essential for maintaining and reestablishing pluripotency. HERV-K can undergo retrotransposition within producer cells without *env* expression, and new integration sites may affect cell proliferation. In induced pluripotent stem cells (iPSCs), genomic impairment due to HERV-K retrotransposition has been identified, but it is a rare event. Considering the retention of SOX2-responsive elements in the HERV-K long terminal repeat (LTR) for over 20 million years, we conclude that HERV-K may play important physiological roles in SOX2-expressing cells.

## INTRODUCTION

Endogenous retroelements are mobile genetic elements that constitute >40% of the human genome. Human endogenous retroviruses (HERVs), encoding long terminal repeat (LTR)-containing elements, account for approximately 8% of the human genome (1–3). For over 20 million years, HERVs that have persisted in germ cell lineages have been vertically transmitted from ancestors to descendants (4). Currently, almost all HERVs have acquired mutations or deletions. However, HERV-K, a relatively new endogenous retrovirus, apparently encodes intact open reading frames (ORFs) in the human genome (5), although no replication-competent HERV-K has been detected (6–9). HERV-K is transcribed during early embryogenesis or exogenous viral infection, producing HERV-K proteins that appear to protect the host cells from viral attack (10–13). HERV-K expression has also been observed in various human diseases, including autoimmune disorders, neurological diseases, infectious diseases, and cancer (14).

Long interspersed nuclear elements (LINE-1), classified as non-LTR retroelements, are transposition competent (15–17). The transposition of LINE-1 occurs mainly in germ cells during early embryonic development. These transposition events may cause pathogenesis by altering the structure, expression, and function of genes (18–20). Therefore, transposition is suppressed by epigenetic mechanisms, including DNA methylation, to prevent harmful mutations in the genome (21, 22). Recent advances in sequencing technology have facilitated the detection of nonreference HERV-K, which is absent from the human genome sequence, in the population (23). However, HERV-K retrotransposition activity has not yet been reported.

HERV-K encodes a 5′ LTR and a 3′ LTR upstream and downstream of the viral protein ORFs, respectively. The HERV-K LTR preserves its promoter activity, and HERV-K is transcribed in embryonic stem cells, cancer cells, and virus-infected T cells (10). The transcription factors Sp1 and Sp3 drive HERV-K transcription in teratocarcinoma cells and melanoma cells (24). The melanoma-specific transcription factor MITF-M is also required to activate the HERV-K LTR (25). In virus-infected cells, the viral transcription factors HIV-1 Tat and human T cell leukemia virus 1 (HTLV-1) Tax are associated with HERV-K expression (26, 27). In embryonic stem cells, DNA hypomethylation and OCT3/4 binding to the HERV-K LTR synergistically facilitate HERV-K transcription (10). In that study, the authors observed that the expression of SOX2 also correlates with HERV-K expression, although they did not examine the importance of SOX2 in HERV-K transcription (10). Therefore, it remains unclear which transcription factors predominantly regulate HERV-K expression.

Here, we show that SOX2, rather than OCT3/4, is the major factor that activates the transcription of HERV-K LTR5Hs, the youngest HERV-K subfamily (5). Consistent with this finding, a large amount of HERV-K Gag was expressed in induced pluripotent stem cells (iPSCs), which are SOX2-expressing cells. As HERV-K movements in the genome in iPSCs were not clarified by whole-genome sequencing, we focused on identifying new HERV-K insertions into numerous reference HERV-K integration sites by using ligation-mediated PCR (LM-PCR) and high-throughput sequencing. The finding of new HERV-K insertions in this study suggests that HERV-K is not a harmless fossil left in the human genome; instead, it retains the ability to spread in the human genome by retrotransposition but is normally repressed because of its dependence on SOX2 expression.

## RESULTS

### SOX2 activates HERV-K transcription.

**FIG 1:**
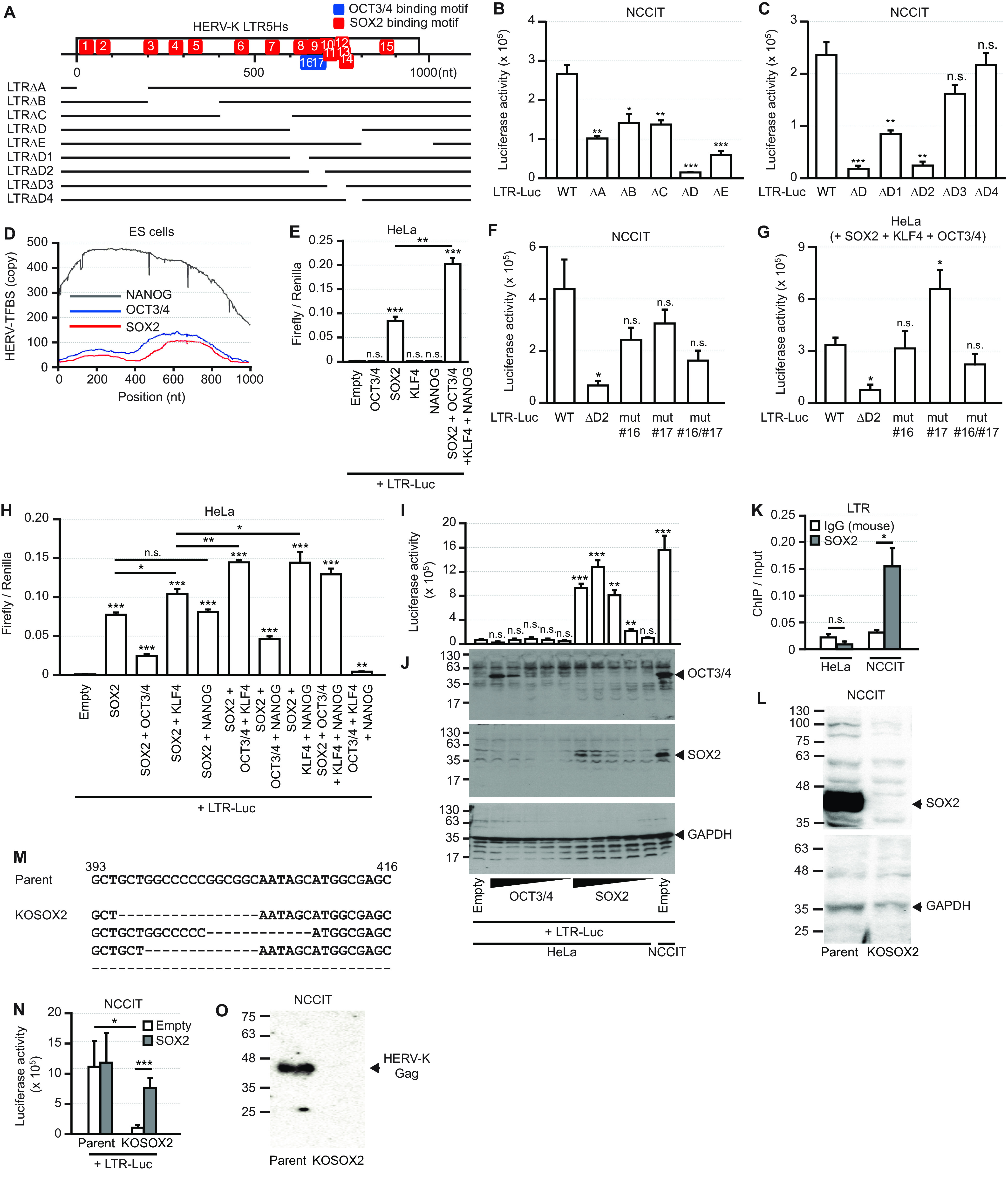
SOX2 contributes to the promoter function of the HERV-K LTR. (A) SOX2- and OCT3/4-binding motifs were identified in the HERV-K LTR by PROMO software, used to identify putative transcription factors. Deletion mutants of the LTR were constructed. (B, C, and F) NCCIT cells were transfected with the pHERV-K_CON_ mutants. The firefly luciferase activities were measured 2 days after transfection by a luciferase reporter assay. (D) Binding sites of SOX2, OCT3/4, and NANOG on the respective LTR5Hs copies in human embryonic stem (ES) cells were collected from the ENCODE ChIP-Seq data set, and the positions on the consensus sequence of LTR5Hs are shown. TFBS, transcription factor-binding site. (E) HeLa cells were cotransfected with the plasmid pHERV-K_CON_ LTR-Luc, the indicated plasmids, and the *Renilla*-Luc plasmid. The firefly and renilla luciferase activities were measured. (G) HeLa cells were cotransfected with pHERV-K_CON_ mutants and the indicated plasmids. The firefly luciferase activities were measured 2 days after transfection by a luciferase reporter assay. (H) HeLa cells were cotransfected with pHERV-K_CON_, the indicated plasmids, and the *Renilla*-Luc plasmid. Two days after transfection, firefly luciferase and *Renilla* luciferase activities were measured by a dual-luciferase reporter assay. (I) HeLa cells were cotransfected with pHERV-K LTR-Luc and different amounts of the indicated plasmids. The luciferase activity was measured. (J) The amounts of OCT3/4, SOX2, and GAPDH proteins were measured by Western blotting. (K) Chromatins in HeLa and NCCIT cells were extracted, and SOX2-binding DNA fragments were immunoprecipitated with the indicated antibodies. HERV-K LTRs in the immunoprecipitated DNA were measured by qPCR. (L) Amounts of SOX2 and GAPDH proteins in NCCIT and SOX2 knockout NCCIT (NCCIT/KOSOX2) cells were measured by Western blotting. (M) The sequences of SOX2 in NCCIT and SOX2 knockout NCCIT (NCCIT/KOSOX2) cells were analyzed. A guide RNA for SOX2 knockout is designed as shown by underlining. (N) NCCIT and NCCIT/KOSOX2 cells were cotransfected with pHERV-K LTR-Luc and SOX2-encoding plasmids. The luciferase activity was measured. For panels B, C, E to I, K, and N, data from three independent experiments are shown as means ± standard deviations. *P* values were determined by Student’s *t* test. *, *P < *0.01; **, *P < *0.001; ***, *P < *0.0001; n.s., not significant. (O) Amounts of mature HERV-K Gag in the supernatants of NCCIT and NCCIT/KOSOX2 cells were measured by Western blotting.

Teratocarcinomas are germ cell tumors, and teratocarcinoma cells constitutively express HERV-K proteins and release HERV-K particles from their plasma membranes (28, 29). Several transcription factors, including MITF, MZF1, NF-Y, GATA-2, and OCT3/4, are required to activate HERV-K LTRs (10, 25, 30). Grow et al. identified consensus OCT3/4-binding motifs in HERV-K LTR5Hs and demonstrated the transcriptional activation of HERV-K by OCT3/4 in human preimplantation embryos (10). However, it is unknown whether the expression of OCT3/4 is sufficient for the transcriptional activation of HERV-K. Here, we identified the region of HERV-K responsible for the transcription of HERV-K LTR5Hs using deletion mutants of HERV-K LTR5Hs in teratocarcinoma (NCCIT) cells (Fig. 1A). These results show that the deletion of nucleotides (nt) 650 to 700 in LTR5Hs causes the loss of transactivation activity (Fig. 1B and C). Using PROMO software (31, 32) to identify putative transcription factors, we identified 15 SOX2-binding motifs (motifs 1 to 15) and 2 OCT3/4-binding motifs (motifs 16 and 17) in the LTR5Hs (Fig. 1A). Two OCT3/4-binding motifs (motifs 16 and 17) and six SOX2-binding motifs (motifs 9, 10, 11, 12, 13, and 14) occurred in the region spanning nt 650 to 700 in LTR5Hs (Fig. 1A). Based on chromatin immunoprecipitation (ChIP) analysis in embryonic stem cells (ENCODE ChIP sequencing [ChIP-Seq] data set), there were two peaks of SOX2 binding at nt 200 and 700 in the HERV-K LTR genome (Fig. 1D). OCT3/4-binding peaks were similar to those of SOX2 in the HERV-K LTR genome. To determine whether OCT3/4 is sufficient for the transactivation of the HERV-K LTR, we cotransfected HeLa cells with plasmids encoding transcription factors (OCT3/4, SOX2, KLF4, and NANOG) and HERV-K LTR-Luc (luciferase) (Fig. 1E). Unexpectedly, we found that OCT3/4 did not activate HERV-K LTR transcription in HeLa cells. The transcription of LTR mutants, which have mutations in the OCT3/4-binding motifs (mutants 16 and 17), was slightly reduced but not significantly so (Fig. 1F and G). In contrast, SOX2 markedly activated HERV-K LTR transcription (Fig. 1E). In the presence of SOX2, KLF4 slightly increased the transactivation of HERV-K, whereas OCT3/4 reduced the effect of SOX2 (Fig. 1H). In the presence of both SOX2 and KLF4, OCT3/4 increased HERV-K transactivation. The transactivation of HERV-K was dose-dependently enhanced by SOX2 expression alone (Fig. 1I and J). However, OCT3/4 alone did not alter the transactivation of HERV-K, even when it was overexpressed.

Because NCCIT cells express large amounts of endogenous SOX2 (Fig. 1J), we examined the binding of endogenous SOX2 to the chromosomal HERV-K LTR using a ChIP assay (Fig. 1K). The results showed that endogenous SOX2 binds to the chromosomal HERV-K LTR in NCCIT cells. To confirm that endogenous SOX2 drives HERV-K transcription, we established SOX2 knockout NCCIT cells (NCCIT/KOSOX2) (Fig. 1L). Although the NCCIT/KOSOX2 genome encodes four different sequence patterns, no intact *SOX2* gene was detected in NCCIT/KOSOX2 cells (Fig. 1M). HERV-K LTR transactivation was dramatically reduced in NCCIT/KOSOX2 cells but not completely lost and was rescued by transfection with SOX2 (Fig. 1N). The mature HERV-K Gag protein (37 kDa) in the viral particles disappeared from the supernatant of the KOSOX2 cells (Fig. 1O). These results indicate that SOX2 is an essential transcription factor for the expression of HERV-K LTR5Hs and that both OCT3/4 and KLF4 drive HERV-K transcription in the presence of SOX2.

### Multiple SOX2-binding motifs activate the HERV-K transcription.

**FIG 2:**
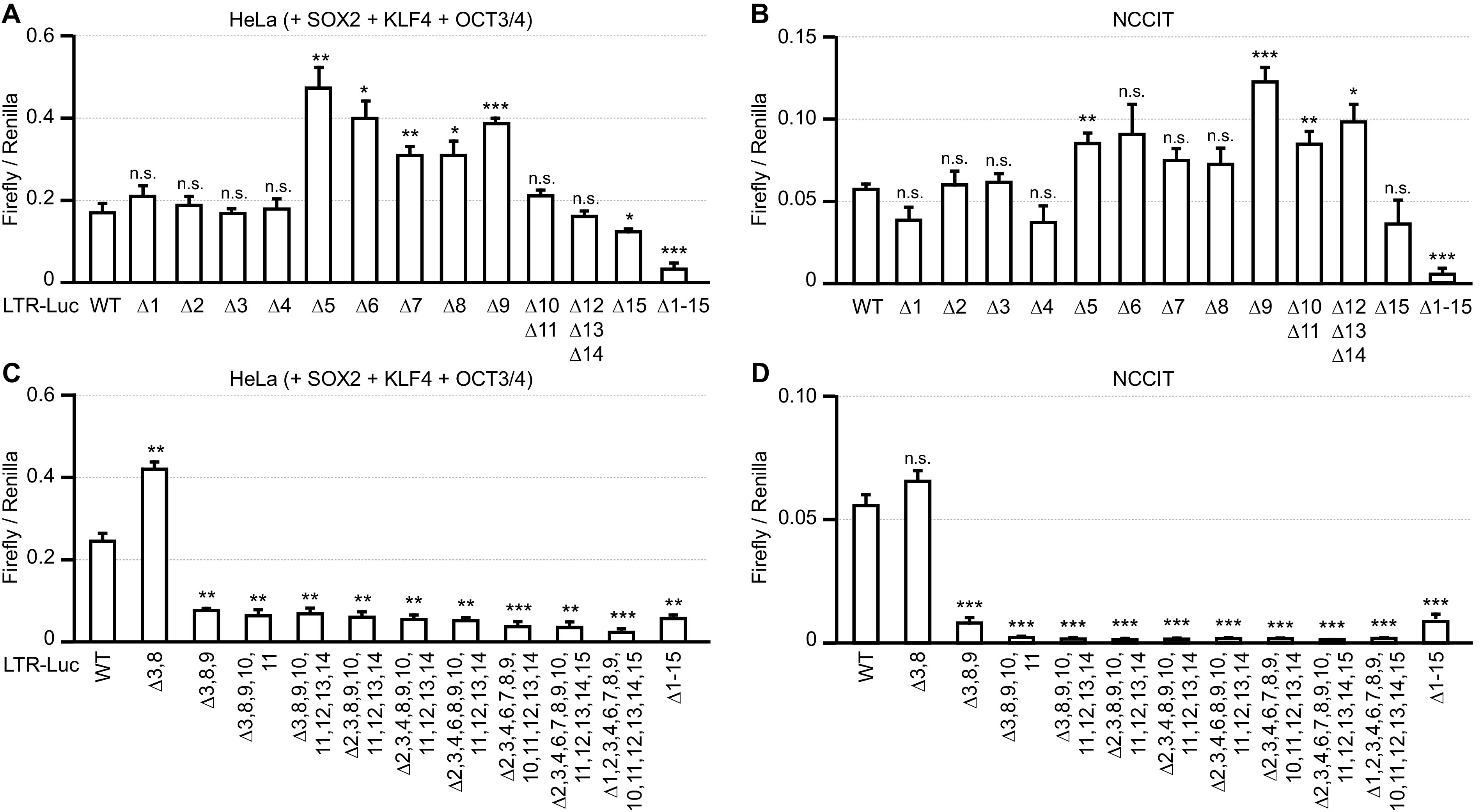
Multiple SOX2-binding motifs contribute to HERV-K transcription. HeLa (A and C) and NCCIT (B and D) cells were cotransfected with pHERV-K LTR mutants, the indicated plasmids, and the *Renilla*-Luc plasmid. The luciferase activity was measured. For panels A to D, data from three independent experiments are shown as means ± standard deviations. *P* values were determined by Student’s *t* test. *, *P < *0.01; **, *P < *0.001; ***, *P < *0.0001; n.s., not significant.

Using PROMO software and the ChIP database, we localized 9 of 15 SOX2-binding motifs (motifs 3, 4, 8, 9, 10, 11, 12, 13, and 14) around nt 200 and 700 in the HERV-K LTR genome (Fig. 1A and D). Based on the data in Fig. 1C, we speculated that the deletion of the single SOX2-binding motif 9 might abolish the transactivation of the HERV-K LTR. HeLa cells were cotransfected with plasmids encoding HERV-K LTR-Luc mutants (deletion mutant 1 [del#1] to del#15) and SOX2, KLF4, and OCT3/4 to determine the region responsible for HERV-K transactivation by SOX2. Unexpectedly, a single deletion of the SOX2-binding motif did not reduce the transactivation of the HERV-K LTR in HeLa cells (Fig. 2A) or NCCIT cells (Fig. 2B). However, the deletion of all SOX2-binding motifs dramatically reduced HERV-K transactivation in HeLa and NCCIT cells. LTR sequences with single deletions showed activity similar to that of the wild-type (WT) HERV-K LTR, but other single deletions enhanced LTR activity, suggesting redundancy and/or interference between SOX2-binding motifs. Therefore, we designed mutants of LTR5Hs with multiple deletions of SOX2-binding motifs (Fig. 2C and D). The deletion of SOX2-binding motifs 3, 8, 9, 10, and 11 around nt 200 and 700 in LTR5Hs, corresponding to two major SOX2-binding regions (Fig. 1A and D), reduced HERV-K transactivation to the same degree as that of the deletion of all SOX2-binding motifs in both HeLa cells (Fig. 2C) and NCCIT cells (Fig. 2D). These results suggest that SOX2 activates HERV-K transcription, even after accumulating several mutations in LTR5Hs during its biological evolution.

### SOX2 activates chromosomal HERV-K expression.

**FIG 3:**
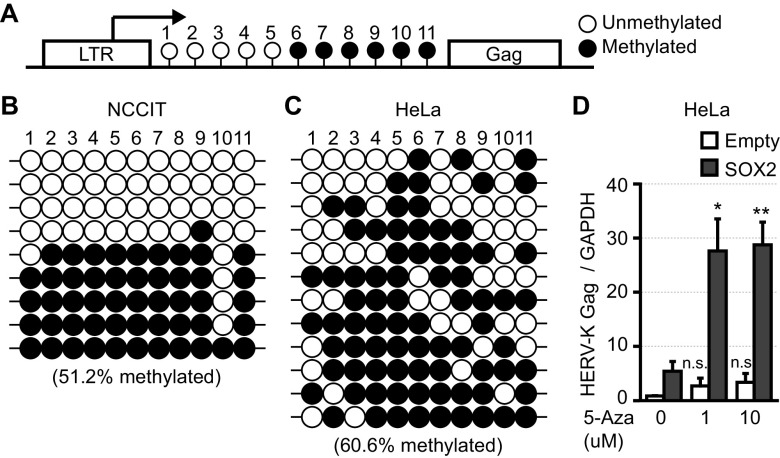
The HERV-K genome is hypermethylated in HeLa cells. (A) There is likely a CpG island (11 CG nucleotides) between the HERV-K LTR and *gag*. (B and C) DNA was extracted from NCCIT (B) and HeLa (C) cells. The sequences of 9 HERV-K genomes between the LTR and Gag in NCCIT cells and 12 HERV-K genomes in HeLa cells were analyzed after the DNAs were treated with bisulfite to convert cytosine residues to uracil. White circles indicate unmethylated nucleotides, and black circles indicate methylated nucleotides in the CpG island. (D) HeLa cells were treated with 5-aza-2′-deoxycytidine (5-Aza) for 1 day and then transfected with a plasmid encoding SOX2. Two days after transfection, the amounts of HERV-K Gag mRNA were measured by RT-qPCR. Data from three independent experiments are shown as means ± standard deviations. *P* values were determined using Student’s *t* test. *, *P < *0.01; **, *P < *0.001; n.s., not significant.

HERV-K genomes have a CpG island between the LTR and *gag* genes (Fig. 3A), hypermethylated in HeLa cells (Fig. 3C) compared to NCCIT cells (Fig. 3B). In NCCIT cells, the three HERV-K clones had unmethylated DNA on the CpG islands (Fig. 3B). This suggests that HERV-K genomes are packed into heterochromatin and are silenced in HeLa cells. We treated HeLa cells with 5-aza-2′-deoxycytidine to demethylate DNA to analyze the effect of DNA methylation on HERV-K expression. Hypomethylation of the genome enhanced HERV-K Gag mRNA expression when SOX2 was overexpressed in HeLa cells (Fig. 3D). These results indicate that DNA hypomethylation and SOX2 expression synergistically induce HERV-K gene expression in the human genome.

### SOX2 activates the 5′ and 3′ LTR5Hs of HERV-K.

**FIG 4:**
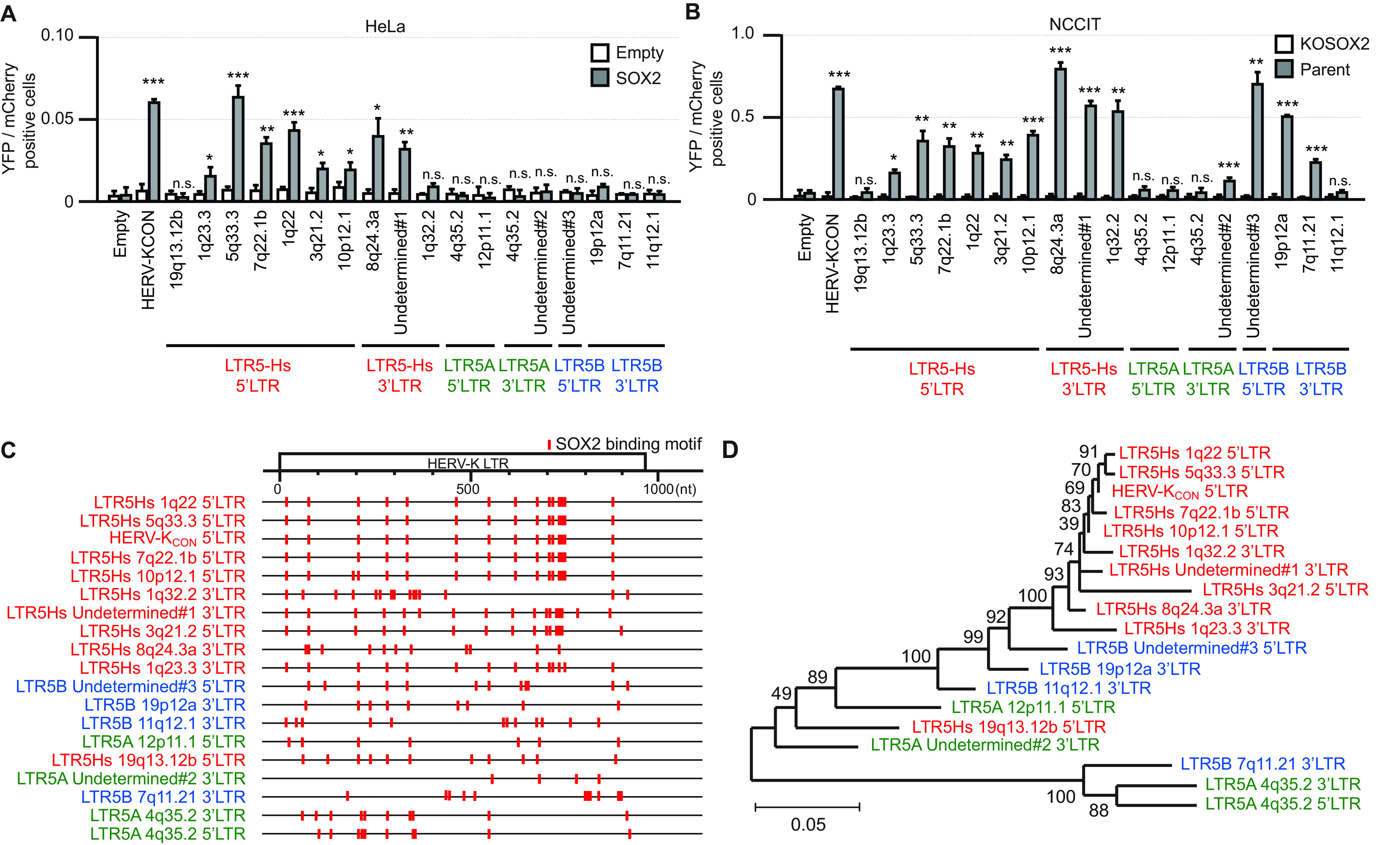
HERV-K transactivation by SOX2 is conserved among HERV-K LTR5Hs. HERV-K LTR series were amplified by PCR from the genome into NCCIT cells and inserted upstream from the yellow fluorescent protein (YFP) gene. (A) HeLa cells were cotransfected with a plasmid encoding SOX2, the pHERV-K LTR-YFP series, and the plasmid encoding red fluorescent protein (mCherry). (B) NCCIT (parent) and NCCIT/KOSOX2 cells were cotransfected with the pHERV-K LTR-YFP series and the plasmid encoding mCherry. In panels A and B, the YFP- and mCherry-positive cells were analyzed by flow cytometry. (C) The locations of SOX2-binding motifs in the HERV-K LTR are shown as red lines. (D) A neighbor-joining tree was constructed based on the aligned nucleotide sequences corresponding to HERV-K LTRs within NCCIT cells.

Because the LTR sequences of HERV-K are classified into three major groups (LTR5Hs, -5A, and -5B), we cloned 18 different HERV-K LTRs from NCCIT cells and investigated whether HERV-K LTR transactivation by SOX2 is conserved among the three different groups. The LTR sequences of the HERV-K 5Hs group (LTR5Hs) are among the most recently integrated sequences (approximately 9.1 million years ago) (33). Two types of LTR5Hs proviruses are classified based on the presence (type 1) or absence (type 2) of a 292-bp deletion at the *pol-env* junction. The LTRs of the 5A and 5B groups (LTR5A and LTR5B) are associated with proviruses, mainly classified as type 2 (33). The LTR5B proviruses include the oldest insertions (around 27.9 million years ago), and LTR5A proviruses (around 20.1 million years ago) originate from LTR5B at an estimated standard mutation rate of 0.24 to 0.45% per million years based on the LTR-based and internally based phylogenies (33). Interestingly, both 5′ and 3′ LTRs of LTR5Hs and three out of four of LTR5B were significantly activated by SOX2, whereas three out of four of LTR5A were not activated in SOX2-expressing HeLa cells (Fig. 4A) and NCCIT cells (Fig. 4B) despite the presence of SOX2-binding motifs in the LTR (Fig. 4C). Phylogenetic analysis of HERV-K LTRs showed that the SOX2-responsive HERV-K LTRs were closely related (Fig. 4D). Both the newest and oldest HERV-K LTRs integrated into genomes retain the capacity for SOX2-dependent transactivation, suggesting that acquiring or maintaining this capacity may be advantageous for the coexistence between HERV-K and the host.

### Reconstructed HERV-K has retrotransposition activity.

**FIG 5:**
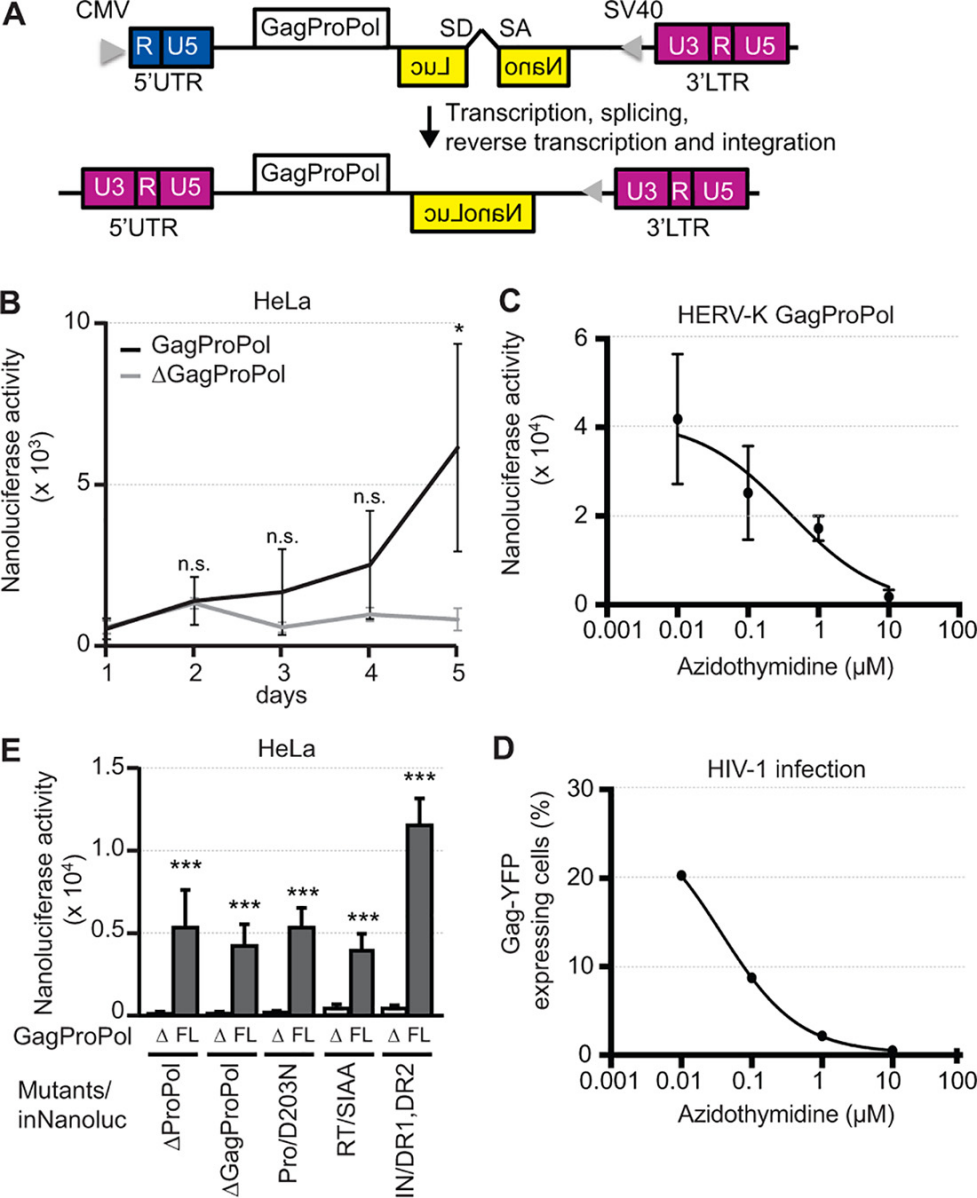
HERV-K has retrotransposition activity in HeLa cells. (A) Construction of pHERV-K GagProPol/inNanoluc. The 5′ U3 region was replaced with the CMV promoter. Intron-disrupted nanoluciferase (inNanoluc) and SV40 were introduced into the Env region in an antisense orientation. SA, splice acceptor site; SD, splice donor site. (B) HeLa cells were transfected with pHERV-K GagProPol/inNanoluc or pHERV-K ΔGagProPol/inNanoluc. Nanoluciferase activity was measured each day by a nanoluciferase reporter assay. (C) HeLa cells were transfected with pHERV-K GagProPol in the presence of azidothymidine. (D) HeLa cells were infected with VSVG-pseudotyped HIV-1 Gag-YFP in the presence of azidothymidine. The percentage of YFP-positive cells was analyzed by flow cytometry. (E) HeLa cells were cotransfected with pHERV-K mutants/inNanoluc and HERV-K ΔGagProPol or HERV-K GagProPol. Five days after transfection, spliced nanoluciferase activity within retrotransposed HERV-K was measured as nanoluciferase activity. Data from six independent experiments (B and E) and four independent experiments (C and D) are shown as means ± standard deviations. *P* values were determined by Student’s *t* test. *, *P < *0.01; **, *P < *0.001; ***, *P < *0.0001; n.s., not significant.

HERV-K LTR5Hs are expressed in SOX2-expressing cells such as germ cells, but it is unclear whether HERV-K has retrotransposition activity within these cells. To examine the retrotransposition activity of HERV-K, we designed an HERV-K_CON_ construct encoding intron-inserting nanoluciferase (inNanoluc) (Fig. 5A). In cells transfected with this construct, pHERV-K GagProPol/inNanoluc, after the transcription of HERV-K from the cytomegalovirus (CMV) promoter, the intact reporter gene sequence was recovered by splicing. If reverse transcription (RT) occurs, the CMV promoter at the 5′ untranslated region (UTR) is replaced with U3 (Fig. 5A, bottom). After integrating the reverse-transcribed HERV-K into the genome, the intact reporter gene was transcribed from the simian virus 40 (SV40) promoter. Therefore, in these experiments, nanoluciferase values indirectly reflected the retrotransposition activity of HERV-K. pHERV-K GagProPol/inNanoluc, which carries full-length *gag*, *pro*, and *pol*, showed nanoluciferase activity 5 days after transfection. In contrast, pHERV-K ΔGagProPol/inNanoluc, which carries only truncated *gag*, showed no significant increase in HeLa cells (Fig. 5B). Nanoluciferase activity was suppressed by the nuclear reverse transcriptase inhibitor (azidothymidine) (Fig. 5C). The antagonistic effect of azidothymidine against HERV-K (50% inhibitory concentration [IC_50_] = 0.388 μM) was lower than that against HIV-1 infection (IC_50_ = 0.037 μM) (Fig. 5D). This suggests that HERV-K protease, reverse transcriptase, and integrase are required for HERV-K retrotransposition. Plasmids encoding HERV-K ΔProPol, a protease mutant (D203N), a reverse transcriptase mutant (SIAA), or an integrase mutant (DR1 and DR2) with the inNanoluc reporter gene were cotransfected into HeLa cells with protein expression plasmids encoding HERV-K ΔGagProPol or HERV-K GagProPol (Fig. 5E). Although these mutants also showed faint nanoluciferase activity, HERV-K GagProPol expression rescued the nanoluciferase activity of these mutants. This indicates that HERV-K protease, reverse transcriptase, and integrase are required to retrotranspose HERV-K. This also suggests that the assembly of intact proteases, reverse transcriptases, and integrases of different HERV-K origins can complement defective HERV-Ks during HERV-K retrotransposition.

**FIG 6:**
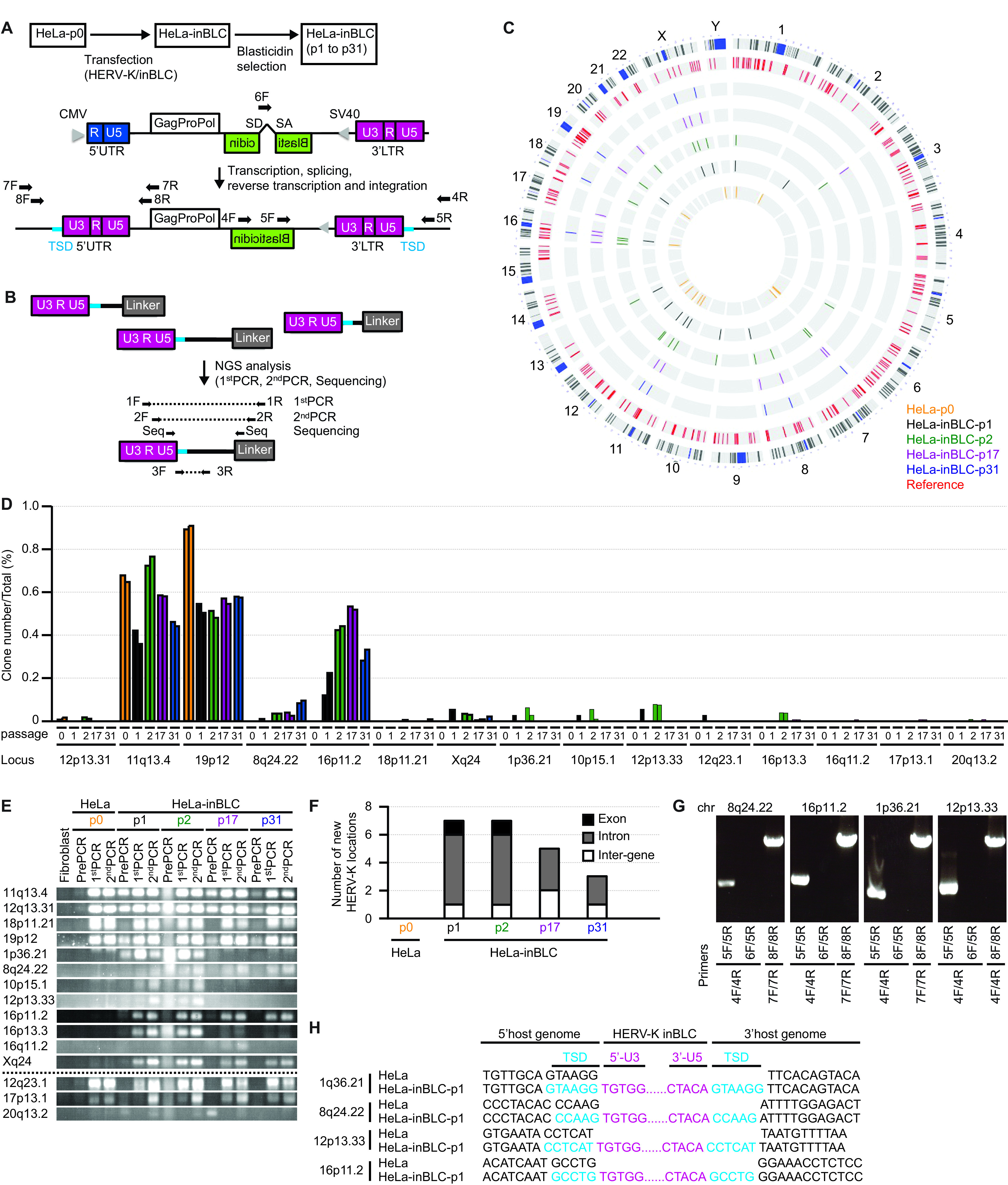
New integration sites for HERV-K appeared in HERV-K-transfected HeLa cells. (A) Construction of pHERV-K GagProPol/inBlasticidin (inBLC). inNanoluc was replaced with inBLC in the Env region of HERV-K. HeLa cells were transfected with pHERV-K inBLC. Blasticidin-resistant cells were selected 2 weeks after transfection. The primers were designed to bind the outside of repeated sequences (7F, 8F, 4R, and 5R) (arrowheads) and the inside of the splicing-out sequence (6F) (arrowheads). (B) The HERV-K DNA in the genome was amplified by PCR and determined by NGS analysis. The primers were designed to bind the 3′ LTRs (1F and 2F) (arrowheads), ligated linkers (1R and 2R) (arrowheads), the region spanning 3′ U5 to the TSD (target-site duplication in the human genome generated by integrase) (3F) (arrowhead), and specific integration sites (3R) (arrowhead). The primers 1F and 2F specifically anneal to the HERV-K LTR but not the murine endogenous retrovirus (MERV) LTR. (C) HERV-K integration sites present in the database are shown in red (Reference). Nonreference HERV-K integration sites in the HeLa and HeLa-inBLC cells, but not fibroblasts, are shown in yellow (passage 0), black (passage 1), green (passage 2), purple (passage 17), and blue (passage 31). The outermost ring is the G band of the human chromosome. (D) NGS analysis detected nonreference HERV-K insertions in HeLa and HeLa-inBLC cells but not fibroblast cells. The copy numbers of each HERV-K integration site were estimated by counting different-length amplicons (34, 35). The total copy number includes both reference and nonreference HERV-K integration sites. (E) HERV-K integration sites in pre-PCR, 1st-PCR, and 2nd-PCR products in HeLa and HeLa-inBLC cells were confirmed by PCR using primers shown in panel B (3F and 3R [arrowheads]). (F) The numbers of specific nonreference HERV-K locations in HeLa and HeLa-inBLC-p1, -p2, -p17, and -p31 cells were determined. (G) HERV-K integration sites (8q24.22, 16p11.2, 1q36.21, and 12p13.33) in HeLa-inBLC-p1 cells were confirmed by PCR. (H) Sequences between the HERV-K LTR and the neighboring HERV-K genomes in HERV-K/inBLC-transfected HeLa cells were analyzed by Sanger sequencing. Corresponding sequences in HeLa cells are also shown for comparison.

To determine the preferred loci for a new integration of HERV-K, we analyzed the new integration sites of HERV-K/inBLC, encoding an intron-containing blasticidin gene in HeLa cells, using ligation-mediated PCR (LM-PCR) (34, 35) to amplify the host-virus junction (Fig. 6A and B; also see Materials and Methods for more details). In the presence of azidothymidine (10 μM) and blasticidin, we could not observe viable cells. This indicates that the blasticidin resistance gene can be reconstituted only by reverse transcription. In the absence of azidothymidine, we identified 311 HERV-K LTR integration sites in the HeLa cell genome. We excluded 48 of the 311 HERV-K LTR integration sites because the HERV-K integration site sequences included deletions or insertions. Of the remaining HERV-K integration sites, 236 sites were present in HeLa and other cell lines, including fibroblasts (see Table S1 in the supplemental material), and 27 sites were present in either HeLa or HeLa-inBLC cells but not fibroblasts (nonreference) (Fig. 6C and Table S2). Using nucleotide BLAST (BLASTn), the Basic Local Alignment Search Tool (BLAST) on the NIH website (https://blast.ncbi.nlm.nih.gov/Blast.cgi), we strictly excluded some HERV-K LTR integration sites that were below 100% identity or had multiple hits within the chromosome database (Table S2). Fifteen of the 27 nonreference HERV-K LTR integration sites were determined to be the exact coordinates of the host genome (Table 1). We also compared these sites in terms of the ratio of each copy number to the total number of integration site copies (Fig. 6D). One of the 15 nonreference integration sites was almost identical to that cited in a previous report (19p12b, K113) (5), while the others have not yet been reported.

**TABLE 1:** Loci of new HERV-K integration sites in HERV-K-transfected HeLa cells

Locus	Coordinate in GRCh38/hg38	Flanking region(s)	Function(s)^*e*^
Universal,^*a*^ HeLa, HeLa-inBLC			
11q13.4	chr11:71800891	FAM86C1 exons 4, 5	
12p13.31	chr12:8231534		
12q23.1	chr12:100854613	L1ME4a^*d*^; ANO4 intron 3, AC138360.1 intron 1	
17p13.1	chr17:8072100		
18p11.21	chr18:15163428	ERVL-MaLRb; AP005901.2 intron 1	
19p12^*c*^	chr19:21658739		
20q13.2	chr20:51642183	AlluSx1^*d*^; ATP9A intron 9	ATPase phospholipid transporting 9A

Specific,^*b*^ HeLa-inBLC			
1p36.21	chr1:15112068	KAZN intron 13, TMEM51-AS1 exon 6	Desmosome assembly, cell adhesion, cytoskeletal organization, and epidermal differentiation
8q24.22	chr8:133275187	NDRG1 intron 3	Stress-responsive protein in hormone responses, cell growth, and differentiation; acts as a tumor suppressor in many cell types
10p15.1	chr10:6263616	LTR50^*d*^	
12p13.33	chr12:768683	WNK1 intron 1	Serine/threonine protein kinases; may be a key regulator of blood pressure by controlling the transport of sodium and chloride ions
16p11.2	chr16:30785310	MIRc^*d*^; ZNF629 intron 1	
16p13.3	chr16:1748182	MAPK8IP3 intron 6	JNK signaling by aggregating specific components of the MAPK cascade
16q11.2	chr16:46398910		
Xq24	chrX:120549077	CUL4B intron 2	Core component of multiple cullin-RING-based E3 ubiquitin-protein ligase complexes

a Universal HERV-K integration sites in HeLa cells.

b Different HERV-K integration sites between HeLa and HERV-K/inBLC-transfected HeLa cells.

c Consistent with a previous report (5).

d The HERV-K-flanking region is in the repetitive sequence.

e JNK, Jun N-terminal protein kinase; MAPK, mitogen-activated protein kinase.

To confirm the presence of new integration events using a different method, we performed PCR using specific primers (3F and 3R) (Fig. 6B) for each integration site and determined the sequences of the amplified products. Eight of the 15 nonreference integration sites were specifically detected in HeLa-inBLC but not HeLa cells (Fig. 6E and Table 1) and were located in introns, exons, or intergenic regions (Fig. 6F). The sequences of these integration sites were confirmed by Sanger sequencing (Table S3) (GenBank accession numbers LC662791 for 11q13.4, LC662792 for 12p13.31, and LC662793 for 18p11.21). Because the primers designed to confirm the three nonreference integration sites (12q23.1, 17p13.1, and 20q13.2) unexpectedly annealed to the reference HERV-K integration sites, the sequences obtained in this analysis were not consistent with the sequences at the coordinates in GRCh38/hg38 based on next-generation sequencing (NGS) analysis (Fig. 6E). Some HERV-K integration sites (8q24.22, 16p11.2, and Xq24) persisted in HeLa-inBLC cells from passage 1 (p1) to passage 31 (p31), whereas other integration sites did not (Fig. 6E). This is consistent with the possibility that HERV-K integration at 8q24.22, 16p11.2, and Xq24 does not cause a growth disadvantage, whereas integration at the other sites does. We designed specific primers for HERV-K/inBLC integration events to further confirm the new HERV-K/inBLC (5F) (Fig. 6A). As expected, amplification products of ∼1,000 to 1,500 bp (5F/5R) and ∼3,000 bp (8F/8R) were observed by nested PCR in HERV-K/inBLC-transfected HeLa cells (Fig. 6G). It is still possible that the sequences of integrated HERV-K/inBLC described above might be sequences integrated into the host genome upon transfection and without reverse transcription. We confirmed the elimination of introns from the BLC sequence at the new HERV-K integration sites to address this possibility. Amplification products of ∼1,500 to 2,000 bp (6F/5R), specific to the intron region in HERV-K/inBLC, were not detected by nested PCR (Fig. 6G). This result suggests that HERV-K/BLC sequences were integrated into the genome after splicing the intron. It is also conceivable that HERV-K retrotransposition depends on the integration machinery through the 3′ poly(A) tail of RNA, similar to LINE-1 (36). However, our sequencing analysis indicated that HERV-K integrase yielded a 5- to 6-bp target-site duplication (TSD), which is conserved in the stably integrated provirus, as in alpha-, beta-, and gammaretroviruses and lentiviruses, but not in LINE-1, in the regions flanking the HERV-K integration sites. Moreover, the CMV promoter at the 5′ LTR was replaced with U3 in each integrated HERV-K by reverse transcription (Fig. 6H and Table S4). According to the sequencing analysis of the entire HERV-K/inBLC at 8q24.22, a persistent integration site, there was no mutation or deletion except for the expected splicing of the intron in the BLC region (Table S4). Although transient expression of the transfected HERV-K/inBLC construct driven by the CMV promoter allows one round of retrotransposition, subsequent retrotransposition does not occur in HeLa cells because the HERV-K LTR is not activated in the absence of SOX2. In summary, these results indicate that reverse-transcribed HERV-K_CON_ genomes are preferentially integrated into introns and intergenes through the retroviral integration machinery, potentially influencing viability depending on the integration sites.

### Endogenous HERV-K retrotransposition occurs in iPSCs.

**FIG 7:**
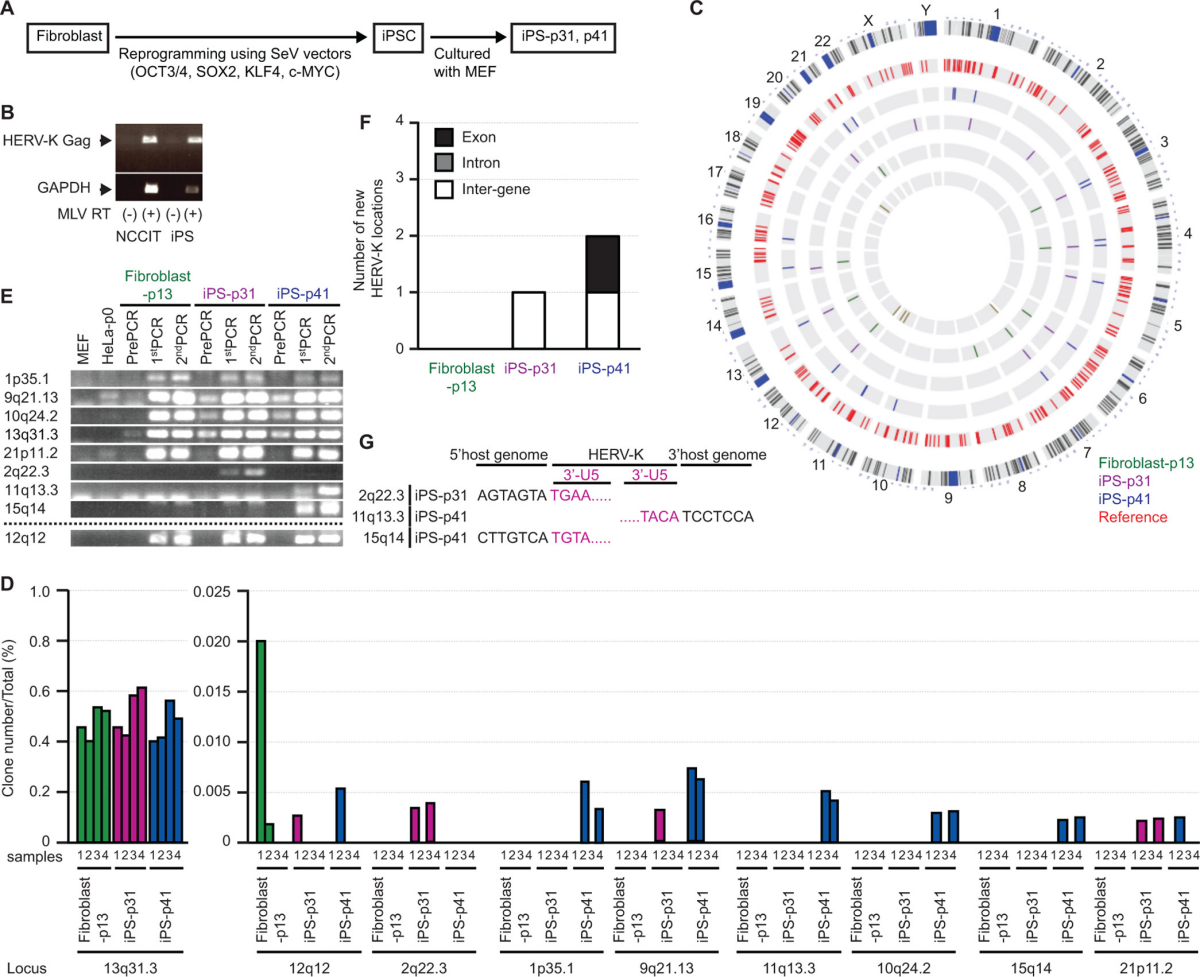
New integration sites for HERV-K appeared in iPSCs. (A) Fibroblast cells were reprogrammed by OCT3/4, SOX2, KLF4, and c-MYC using Sendai virus (SeV) vectors. iPSCs were cocultured with MEFs for 31 passages (iPS-p31) and 41 passages (iPS-p41), and the HERV-K DNA in the genome was then amplified by PCR and determined by NGS analysis. (B) HERV-K Gag mRNA and GAPDH mRNA expression levels in NCCIT cells and iPSCs were measured by reverse transcription-PCR. (C) Integration sites of HERV-K were determined by NGS analysis. HERV-K integration sites already present in the database (Reference) are shown in red. Nonreference HERV-K integration sites in fibroblasts and iPSCs but not HeLa cells are shown in green (fibroblasts), purple (iPS-p31 cells), and blue (iPS-p41 cells). (D) NGS analysis detected nonreference HERV-K insertions in fibroblasts and iPSCs but not HeLa cells. The copy numbers of each HERV-K integration site were estimated by counting different-length amplicons (34, 35). The total copy number includes both reference and nonreference HERV-K integration sites. (E) HERV-K integration sites in pre-PCR, 1st-PCR, and 2nd-PCR products of each HeLa cell were confirmed by PCR (3F and 3R [arrowheads] in panel A). (F) The number of specific nonreference HERV-K locations in iPSCs was determined. (G) Sequences between the HERV-K LTR and the neighboring HERV-K genomes in iPS-p31 or iPS-p41 cells were analyzed by Sanger sequencing.

Recently, iPSCs have become potential research models for regenerative medicine. Fibroblasts were reprogrammed by at least three factors, SOX2, OCT3/4, and KLF4 (Fig. 7A), to develop iPSCs. Therefore, we speculated that HERV-K expression may be induced by SOX2 in iPSCs. As expected, HERV-K Gag mRNA was detected in both NCCIT cells and iPSCs (Fig. 7B), indicating that unregulated HERV-K might be transposed in the genomes of iPSCs. To investigate HERV-K retrotransposition, we analyzed HERV-K integration sites in fibroblasts and iPSCs from the same donor (Fig. 7C and Table S5). In this analysis, we excluded uncertain integration sites using the Basic Local Alignment Search Tool on the NIH website (Table S5). Nine of the 30 nonreference HERV-K LTR integration sites were determined as exact coordinates on the host genome (Table 2) and were compared for the ratio of each copy number to the total number of integration site copies (Fig. 7D). We found four nonreference HERV-K insertions shared between the fibroblasts and iPSCs but not HeLa cells (Fig. 7E and Table 2) (universal in fibroblasts and iPSCs). Two of the four nonreference HERV-K insertions were consistent with previously reported HERV-K integration sites (12q12, K20; 13q31.3, K22) (37). Other nonreference HERV-K insertion sites (1p35.1 and 10q24.2) may be unique individual-specific HERV-K sites. The sequences of these integration sites were confirmed by Sanger sequencing (Table S6) (GenBank accession numbers LC662794 for 1p35.1 and LC662795 for 10q24.2). Notably, we found three nonreference, “iPSC-specific” HERV-K integration sites, which were detected in iPSCs but not the parental fibroblast cells (Fig. 7E and F and Table 2) (specific in iPSCs). We confirmed three iPSC-specific integration sites in the 1st and 2nd PCR products from LM-PCR (Fig. 7E). The virus-host junctions of these integration sites were at the end of the LTR 3′ U5. The DNA strand orientation was antisense for two of the three integration sites (2q22.3 and 15q14) (Fig. 7G). This suggests that these integration sites were formed by retrotransposition or reinfection of HERV-K rather than genomic rearrangements or technical artifacts in PCR. Of the three iPSC-specific HERV-K insertions, two insertions were found in the intergenic regions. At the same time, the other was in the UTR of an exonic region (Fig. 7F and Table 2) (specific to iPSCs). These results suggest that HERV-K has retrotransposition activity in iPSCs. However, we found no continuous nonreference HERV-K integration site for long-term culture of iPSCs (Fig. 7C), suggesting that clonal expansion is rare in iPSCs.

**TABLE 2:** Loci of new HERV-K integration sites in iPSCs

Locus	Coordinate in GRCh38/hg38	Flanking region(s)	Function
Universal,^*a*^ fibroblast, iPS-p31, iPS-p41			
1p35.1	chr1:32614846	AluSc^*d*^	
10q24.2	chr10:99256369	MSTD int^*d*^	
12q12^*c*^	chr12:43919854	L1MB1^*d*^; TMEM117 intron 2	
13q31.3^*c*^	chr13:90090934	LINC00559 intron 3	Long noncoding RNA

Specific,^*b*^ iPS-p31, iPS-p41			
2q22.3	chr2:147700152		
11q13.3	chr11:70471356	SHANK2 exon 2	Molecular scaffolds in the postsynaptic density of excitatory synapses (autism susceptibility 17, autism spectrum disorder, pervasive developmental disorder, deafness, autosomal recessive 63, secretory diarrhea, autistic disorder)
15q14	chr15:36348199		

a Universal HERV-K integration sites in this donor.

b Different HERV-K integration sites between fibroblasts and iPSCs.

c Consistent with a previous report from Robert Belshaw’s group (37).

d HERV-K flanking region is in the repetitive sequence.

## DISCUSSION

In this study, we demonstrated that HERV-K could retrotranspose in SOX2-expressing cells. The transactivation of HERV-K LTR5Hs and LTR5B by SOX2 was retained, even after the accumulation of several mutations in these LTR sequences. Although the physiological roles of HERV-K are still unknown, we found that HERV-K exhibits retrotransposition activity and moves randomly around the host genome. In a blasticidin-selected cell population where SOX2 was not expressed and, hence, retrotransposition occurred only once after transfection, the copy number of HERV-K, which is integrated into the intron of a tumor suppressor gene (*NDRG1*), increased in passage 31. This suggests that HERV-K integration rarely cancels cell death or cell growth interruption by impairing the host genome and, thus, might be sustained for long-term culturing. The impairment of the host genome can cause several diseases (discussed below). However, in SOX2-expressing cells, only a small number of novel HERV-K integration sites were identified. SOX2-expressing cells with a new integration of HERV-K may die or grow slowly during long-term culture because of the harmful impact of HERV-K integration on genome integrity.

In addition to its well-known role in the maintenance and reestablishment of pluripotency (38, 39), SOX2 is essential for central nervous system (CNS) development and the maintenance of neural stem cells (40). SOX2 is also expressed in Schwann cells (41) and impairs Schwann cell remyelination and functional recovery after nerve injury, as in multiple sclerosis (42). Therefore, it is conceivable that SOX2-induced expression of HERV-K might impact CNS development, maintenance of neural stem cells, remyelination, or recovery from nerve injury. HERV-K is implicated in several neural diseases, including multiple sclerosis (43). Moreover, HERV-K LTR integration sites differ slightly among the genomes of individual humans and between human tissues, and HERV-K LTR single-nucleotide polymorphisms (SNPs) are implicated in several neural diseases (44). It is possible that SOX2 influences the expression of genes adjacent to HERV-K LTR5Hs. Additionally, our results are consistent with the possibility that HERV-K expression, which becomes uncontrollable when the epigenetic regulation of SOX2 is disturbed, disrupts the nervous system through the retrotransposition of HERV-K.

SOX2 is associated with numerous cancers (45). It regulates the self-renewal and maintenance of cancer stem cell populations by promoting oncogenic signaling (46–48). HERV-K expression is considerably higher in malignant tissues such as germ cell tumors, melanomas, and ovarian cancers than in healthy tissues (49–52), suggesting that the transactivation of HERV-K LTR5Hs by SOX2 is involved in numerous malignant tumors. Whether HERV-K expression is involved in the self-renewal and maintenance of cancer stem cells is still unknown, but the impairment of the genome by HERV-K retrotransposition may cause malignancy in tumor tissues.

HERV-K is transiently reactivated in early human development to protect cells from the threat of exogenous viral infection (10); however, HERV-K retrotransposition entails a risk of genomic impairment in SOX2-expressing cells, such as iPSCs. According to our results, such genomic impairment is probably a rare event in iPSCs. In addition to the possibility that HERV-K retrotransposition causes a defect in cell proliferation, thereby reducing cells with genomic impairment, HERV-K retrotransposition may be prohibited by host restriction factors during reverse transcription and/or integration. For example, APOBEC3F, a restriction factor in cell-free HERV-K infection (9), may inhibit HERV-K retrotransposition during reverse transcription. In the yeast Saccharomyces cerevisiae, Ty1 LTR retrotransposon Gag forms virus-like particles as retrosomes for reverse transcription (53). APOBEC3G interacts with Ty1 Gag in retrosomes and restricts Ty1 retrotransposition (54, 55). However, it is unknown whether HERV-K Gag forms a retrosome similar to Ty1 (53) or whether APOBEC3F can access the HERV-K genome in the retrosome. In the future, the mechanism of HERV-K retrotransposition should be clarified.

Transposable elements, such as HERVs, often provide new functions to vertebrate hosts, resulting in exaptation (56). Endogenous retrovirus Env, also known as syncytin, is necessary for the fusion of cytotrophoblasts to form the multinucleate syncytiotrophoblast layer of the placenta (57). Syncytins are involved in convergent evolution during the transition from oviparity to viviparity because syncytins originate independently across multiple mammalian lineages and a live-bearing reptile (58, 59). Additionally, the neuronal gene *Arc*, a retrotransposon Gag protein, mediates intercellular signaling in neurons and is essential for cognition in animals (60). This suggests that the retrotransposon Gag has obtained alternative functions in neurons during evolution. The function of HERV-K remains unclear, but considering the acquisition of SOX2-responsive elements and the retention of competent elements in their LTRs, we can conclude that HERV-K may play important physiological roles in restricted SOX2-expressing cells to minimize the risk of movements in the genome. Further study to unveil the mechanism of HERV-K retrotransposition, a rare movement in iPSCs, would be an important finding for the development of regenerative medicines.

## MATERIALS AND METHODS

### Plasmids.

Full-length HERV-K_CON_ was kindly provided by Paul Bieniasz (9). pHERV-K_CON_ LTR-Luc carries the luciferase gene driven by the HERV-K LTR. pMXs-SOX2, -OCT3/4, -KLF4, and -NANOG were purchased from Addgene. CHKCinNluc and CHKCinBLC were derived from CHKCP (kindly provided by Paul Bieniasz [9]). The puromycin *N*-acetyltransferase gene was removed from CHKCP (CHKCP/ΔPuro), and a NotI site was inserted. Intron-disrupted nanoluciferase (inNanoluc) and blasticidin (inBLC) were designed as previously described (61). The inNanoluc and inBLC cassettes encoded the SV40 early enhancer/promoter and SV40 late poly(A) signals, respectively. These cassettes were introduced into CHKCP/ΔPuro at the NotI site in an antisense orientation.

### Cells.

HeLa cells were cultured in Dulbecco’s modified Eagle’s medium (DMEM; Sigma-Aldrich) supplemented with 5% fetal bovine serum (FBS). NCCIT cells (ATCC CRL-2073) were cultured in RPMI 1640 medium with 10% FBS, 1 mM sodium pyruvate, and GlutaMAX (62). Human iPSCs were generated from human skin-derived fibroblasts and cultured with mitomycin C-treated mouse embryonic fibroblast feeder cells (MEFs), as previously described (63–65).

### Isolation of skin-derived fibroblasts.

All experimental procedures involving human fibroblasts were approved by the following Kumamoto University ethics committees: Epidemiological and General Research at the Faculty of Life Science, Human Genome and Gene Analysis Research at the Faculty of Life Sciences, and Clinical Research and Advanced Medical Technology (approval numbers 318, 153, and 1018, respectively). The healthy volunteer in this study received a verbal explanation of the procedures and provided written informed consent. Human skin-derived fibroblasts were generated and isolated from the explant of a skin biopsy specimen collected from a healthy volunteer. The skin sample was minced and cultured in DMEM (Thermo Fisher Scientific) supplemented with 10% FBS.

### Generation of NCCIT/KOSOX2 cell lines.

Lentiviral particles were harvested by cotransfection of 293FT cells with guide RNA-carrying lentiCRISPR v2, a packaging plasmid (psPAX2), and an envelope vector expressing the vesicular stomatitis virus glycoprotein (VSVG) (pMD2.G) using Lipofectamine 3000 (Invitrogen). A 20-bp guide sequence (5′-GCTCGCCATGCTATTGCCGC-3′) targeting a DNA sequence was designed using the CRISPOR program (66). The lentiviral particles were transduced into NCCIT cells. The transduced cells were selected in the presence of 1 μg/mL puromycin and cloned by limiting dilution.

### HERV-K retrotransposition assay.

HeLa cells were seeded into six-well plates at a density of 2 × 10^5^ cells/well. The cells were transfected with Lipofectamine 3000 reagent (Invitrogen), according to the manufacturer’s protocol. The cells were harvested 1 to 6 days after transfection, and the nanoluciferase activity in the cells was measured using the Nano-Glo luciferase assay reagent (Promega).

### Measurement of dual-luciferase luminescence.

Luminescence was measured using the dual-luciferase reporter assay system (Promega) according to the manufacturer’s instructions. The cell lysate was mixed with luciferase assay reagent II. The firefly luciferase activity was measured using a luminometer. *Renilla* luciferase activity was measured after mixing the cell lysate containing luciferase assay reagent II with Stop & Glo reagent.

### Bisulfite sequencing.

The EpiTect Plus bisulfite conversion kit (Qiagen) was used, as previously described (10). PCR fragments were inserted into the pCR-BluntII-Topo vector (Invitrogen). Approximately 10 clones from HeLa and NCCIT cells were sequenced to quantify CpG methylation.

### ChIP assay.

NCCIT cells were fixed with 1% formaldehyde and lysed with 20% NP-40 (10 mM HEPES-KOH [pH 7.9], 1.5 mM MgCl_2_, 10 mM KCl, 0.5 mM dithiothreitol [DTT], and 20% NP-40 with a protease inhibitor cocktail [Roche]). Chromatin in the lysates was fragmented to 320 bp after digestion with micrococcal nuclease. After further lysis with 10% SDS (50 mM Tris-HCl [pH 8.1], 0.2 mM EDTA, 10% SDS, and a protease inhibitor cocktail [Roche]), the chromatin was sonicated. The DNA-protein complexes were precipitated overnight by incubation with an anti-SOX2 antibody (BioLegend) and then incubated with ChIP-grade protein G magnetic beads (catalog number 9006; Cell Signaling Technology) for 2 h. The abundance of the HERV-K LTR in the precipitated DNA was analyzed by quantitative PCR (qPCR) using HERV-K LTR forward (F) primer 5′-AGCACTGAGATGTTTATGTG-3′ and reverse (R) primer 5′-TGTGGGGAGAGGGTCAGC-3′ and SYBR Premix Ex *Taq* II (TaKaRa Bio Inc.). The signal intensity was quantified using an ABI 7900HT Fast real-time PCR system (Applied Biosystems).

### Ligation-mediated PCR.

HERV-K integration sites were analyzed by ligation-mediated PCR (LM-PCR) and high-throughput sequencing, as previously described, with minor modifications (34, 35). To analyze the HERV-K integration site, the junction between the 3′ LTR of HERV-K and the host genomic DNA was amplified with a primer targeting the 3′ LTR and the linker. The first forward primer targeting the 3′ LTR was B3-K1 (5′-CCTCCATATGCTGAACGCTGGTT-3′); the second forward primer targeting the 3′ LTR was P5B5-K2 (5′-AATGATACGGCGACCACCGAGATCTACACCCAAATCTCTCGTCCCACCTTACGAGAAACACCCACAGG-3′). DNA libraries were sequenced as paired-end reads using the Illumina MiSeq platform, and the resulting FASTQ files were analyzed. The sequencing primer targeting the 3′ LTR was Seq-K1 (5′-ACACCCACAGGTGTGTAGGGGCAACCCACC-3′). The flanking host genome sequences were used to determine the viral integration sites. To increase the accuracy of the demultiplexing step, we used an in-house script that extracts only reads with a high index read sequencing quality (Phred score of >20) from each position of an 8-bp index read. Sequencing reads containing the 3′ ends of HERV-K LTR sequences (CCTACA and CCTTCA) were selected, the LTR sequences were trimmed, and the remaining reads without the HERV-K sequence were used for mapping the human genome using the BWA-MEM algorithm (67). Further data processing and cleanup, including removing reads with multiple alignments and duplicated reads, were performed using Samtools (67) and Picard (http://broadinstitute.github.io/picard/). Universal and specific integration sites in HeLa cells, fibroblasts, and iPSCs were identified using the Excel COUNTIFS tool. The sequences of selected integration sites were manually verified with the human GRCh38/hg38 database using the UCSC genome browser and the Basic Local Alignment Search Tool on the NIH website.

### Western blotting.

Cells and viruses were lysed with 1% Triton X lysis buffer (50 mM Tris-HCl [pH 7.5] containing 0.5% Triton X-100, 300 mM NaCl, 10 mM iodoacetamide, and a protease inhibitor cocktail [Roche]). After 2× SDS sample buffer was added, the SOX2, OCT3/4, glyceraldehyde-3-phosphate dehydrogenase (GAPDH), and HERV-K Gag proteins were detected by immunoblotting using anti-SOX2 antibody (Merck Millipore), anti-OCT3/4 antibody (BD Biosciences), anti-GAPDH antibody (Sigma), and anti-HERV-K Gag antibody (Austral Biologicals), respectively, as the primary antibodies. Horseradish peroxidase (HRP)-conjugated anti-mouse IgG antibody (Jackson ImmunoResearch) was used as a secondary antibody. HRP-conjugated secondary antibody was detected using Chemi-Lumi One L (Nacalai Tesque).

### Reverse transcription-quantitative PCR analysis.

Total RNA was purified using an RNeasy minikit (Qiagen, Hilden, Germany). The mRNA was reverse transcribed with murine leukemia virus (MLV) reverse transcriptase after annealing with a poly(T) primer. HERV-K *gag* DNA was amplified with the following primers: HERV-K Gag CA forward primer 5′-CAAGACCCAGGAAGTACCT-3′ and reverse primer 5′-ACACTCAGGATTGGCGTT-3′. All qPCR assays were performed using SYBR Premix Ex *Taq* II (TaKaRa Bio Inc.). The data for the target genes were then normalized to the expression level of the GAPDH housekeeping gene and amplified with GAPDH forward primer 5′-CGCTCTCTGCTCCTCCTGTT-3′ and reverse primer 5′-ACAAAGTGGTCGTTGAGGGC-3′.

### Cloning the HERV-K LTRs.

Total DNA was extracted from NCCIT cells using a DNeasy blood and tissue kit (Qiagen). The HERV-K LTR was amplified with HERV-K LTR primers (5Hs forward primer 5′-CCAAAAGCCATCGATTGTGGGGAAAAGCAAGAGAG-3′, 5Hs 5′-LTR reverse primer 5′-TTCCATCTCGAGTGAAGTGGGGCCAGCCCCTCCACACCT-3′, 5Hs 3′-LTR reverse primer 5′-TTCCATCTCGAGTGTAGGGGTGGGTTGCCCCTCCACACC-3′, 5A forward primer 5′-AAAGCCATCGATTGTAGGGAAAAGAAAGAGAGATCAGAC-3′, 5A 5′-LTR reverse primer 5′-TTCCATCTCGAGTGAAGGGGTGGCCTGCCCCTCCA-3′, 5A 3′-LTR reverse primer 5′-TTCCATCTCGAGCTCCACACCTGTGGGTAT-3′, 5B forward primer 5′-AAAGCCATCGATTGTAGGGAAAAGAAAGAGAGATCAG-3′, 5B 5′-LTR reverse primer 5′-TTCCATCTCGAGTGAAGTGGGGCCAGCCCCTCCACACCT-3′, and 5B 3′-LTR reverse primer 5′-TTCCATCTCGAGCTCCACACCTGTGGGTATTTCT-3′).

### Flow cytometry analysis.

The HERV-K LTR was inserted upstream of the yellow fluorescent protein (YFP)-encoding gene (HERV-K LTR-Venus). HeLa and NCCIT cells were cotransfected with pHERV-K LTR-Venus and pMXs-SOX2. Two days after transfection, fluorescence signals were analyzed using flow cytometry.
