# Supplementary material for: Movements of Ancient Human Endogenous Retroviruses Detected in SOX2-Expressing Cells
Source: J Virol. 2022 Apr 14;96(9):e00356-22. doi: 10.1128/jvi.00356-22 (PMC9093106; doi:10.1128/jvi.00356-22)
Supplement: Supplemental file 1 — Tables S1 to S6. Download jvi.00356-22-s0001.pdf, PDF file, 0.1 MB [file jvi.00356-22-s0001.pdf]

Supplemental Tables

Table S1. Loci of HERV-K integration sites in both HeLa and fibroblast cells.
Table S2. Loci of new HERV-K integration sites in HeLa and HERV-K-transfected HeLa cells.
Table S3. Sequences of new HERV-K integration sites in HERV-K-transfected HeLa cells.
Table S4. The sequence of HERV-K/BLC genome with integration sites at 8q24.22 in HERV-K-transfected HeLa cells.
Table S5. Loci of new HERV-K integration sites in fibroblast and iPS cells.
Table S6. Sequences of new HERV-K integration sites in iPS cells.

References

1. Subramanian RP, Wildschutte JH, Russo C, Coffin JM. 2011. Identification, characterization, and comparative genomic distribution of the HERV-K (HML-2) group of human endogenous retroviruses. Retrovirology 8:90.
2. Barbulescu M, Turner G, Seaman MI, Deinard AS, Kidd KK, Lenz J. 1999. Many human endogenous retrovirus K (HERV-K) proviruses are unique to humans. Curr Biol 9:861-8.
3. Costas J. 2001. Evolutionary dynamics of the human endogenous retrovirus family HERV-K inferred from full-length proviral genomes. J Mol Evol 53:237-43.
4. Marchi E, Kanapin A, Magiorkinis G, Belshaw R. 2014. Unfixed endogenous retroviral insertions in the human population. J Virol 88:9529-37.
5. Ono M, Yasunaga T, Miyata T, Ushikubo H. 1986. Nucleotide sequence of human endogenous retrovirus genome related to the mouse mammary tumor virus genome. J Virol 60:589-98.
6. Hughes JF, Coffin JM. 2001. Evidence for genomic rearrangements mediated by human endogenous retroviruses during primate evolution. Nat Genet 29:487-9.
7. Sugimoto J, Matsuura N, Kinjo Y, Takasu N, Oda T, Jinno Y. 2001. Transcriptionally active HERV-K genes: identification, isolation, and chromosomal mapping. Genomics 72:137-44.
8. Medstrand P, Mager DL. 1998. Human-specific integrations of the HERV-K endogenous retrovirus family. J Virol 72:9782-7.
9. Lee E, Iskow R, Yang L, Gokcumen O, Haseley P, Luquette LJ, 3rd, Lohr JG, Harris CC, Ding L, Wilson RK, Wheeler DA, Gibbs RA, Kucherlapati R, Lee C, Kharchenko PV, Park PJ, Cancer Genome Atlas Research N. 2012. Landscape of somatic retrotransposition in human cancers. Science 337:967-71.
10. Tonjes RR, Czauderna F, Kurth R. 1999. Genome-wide screening, cloning, chromosomal assignment, and expression of full-length human endogenous retrovirus type K. J Virol 73:9187-95.
11. Turner G, Barbulescu M, Su M, Jensen-Seaman MI, Kidd KK, Lenz J. 2001. Insertional polymorphisms of full-length endogenous retroviruses in humans. Curr Biol 11:1531-5.
12. Wildschutte JH, Williams ZH, Montesion M, Subramanian RP, Kidd JM, Coffin JM. 2016. Discovery of unfixed endogenous retrovirus insertions in diverse human populations. Proc Natl Acad Sci U S A 113:E2326-34.
13. Belshaw R, Dawson AL, Woolven-Allen J, Redding J, Burt A, Tristem M. 2005. Genomewide screening reveals high levels of insertional polymorphism in the human endogenous retrovirus family HERV-K(HML2): implications for present-day activity. J Virol 79:12507-14.
14. Macfarlane C, Simmonds P. 2004. Allelic variation of HERV-K(HML-2) endogenous retroviral elements in human populations. J Mol Evol 59:642-56.

Table S1. Loci of HERV-K integration sites in both HeLa and fibroblast cells.

Coordinate GRCh38/hg38	Clone number / Total (%)[a]	Coordinate GRCh38/hg38	Clone number / Total (%)[a]	Coordinate GRCh38/hg38	Clone number / Total (%)[a]
**Reference**					
chr12:51454286	1.297	chr21:43147129	0.754	chr1:27189148	0.006
chr8:139459905 [b]	1.278	chr5:156657705 [f]	0.751	chr11:72165521	0.006
chr1:155626665 [c]	1.247	chr13:19601186	0.689	chr17:68603991	0.006
chr12:32100479	1.232	chr1:77982895	0.630	chr5:169997721	0.006
chr20:41971864	1.195	chr22:18947849 [c]	0.621	chr15:83997549	0.006
chr9:131357677	1.195	chr3:185570759 [g]	0.614	chr12:83447521	0.006
chr3:47260502	1.191	chr11:63530313	0.580	chr22:24210814	0.006
chr3:113024276 [c]	1.191	chr3:148567609 [b]	0.568	chr19:51908191	0.006
chr1:93276781	1.188	chr1:66424814	0.568	chr1:160691754 [c]	0.006
chr2:27460947	1.188	chr3:185562547 [g]	0.558	chr16:34999772 [b]	0.006
chr7:125221169	1.179	chrX:90951584	0.558	chr8:143849485	0.006
chr10:26894438 [c]	1.173	chr7:104748889 [b]	0.540	chr1:209142225	0.006
chr9:95269770	1.167	chr9:31632878	0.537	chr9:134092391	0.006
chr3:195927524	1.167	chr2:231577744	0.534	chr7:16197551	0.006
chr11:101704529 [d]	1.160	chr3:101692862 [h]	0.527	chr13:49924057	0.006
chr8:54031680	1.151	chr1:75383459 [g]	0.527	chr17:54202542	0.006
chr7:4582425 [c]	1.145	chr4:160659754 [b]	0.521	chr17:30699739	0.006
chr2:37226286	1.139	chrY:10119161	0.521	chr1:77982751	0.006
chr6:77716944 [c]	1.136	chr11:118729206 [d]	0.509	chr1:209142174	0.006
chr12:9601433	1.123	chr17:30700040	0.506	chr22:24210698	0.006
chr22:18939642 [c]	1.111	chr12:110571006 [i]	0.500	chr1:33064485	0.003
chr5:75605832	1.108	chr12:6885859	0.487	chr17:5074267	0.003
chr19:37332373	1.092	chr1:111259975 [j]	0.478	chr18:69042851	0.003
chr16:47865427	1.089	chr5:8938709	0.468	chr12:118107118	0.003
chr15:88541520	1.083	chr1:224340799	0.394	chr10:42109357	0.003
chr5:179513962	1.083	chr10:43338171	0.363	chr2:16234327	0.003
chr3:14091184	1.070	chr8:90685847	0.344	chr5:169996874	0.003
chr7:123781605	1.064	chr6:160849867 [e]	0.335	chr17:80551024	0.003
chr5:1595976	1.064	chr12:58335947 [c]	0.332	chr15:66223683	0.003
chrX:125780479	1.064	chr1:1410774	0.310	chrX:145721687	0.003
chr9:121430074	1.061	chr8:43739538	0.292	chr3:186894450	0.003
chr4:72129223	1.055	chr8:57199602	0.279	chr2:6187757	0.003
chr14:64978587	1.049	chr11:72165342	0.273	chr15:63082400 [e]	0.003
chr6:88382556	1.049	chr3:50520849	0.273	chr2:232423966	0.003
chr19:37867145	1.046	chr11:101696031 [d]	0.233	chr9:133958742	0.003
chr10:41713154	1.030	chr5:156665917 [f]	0.226	chr3:113024210 [c]	0.003
chr4:151741	1.024	chr7:4599432 [c]	0.208	chr7:16198773	0.003
chr18:2000813	1.024	chr9:17446329	0.186	chr6:160849956	0.003
chr5:35177354	1.018	chr3:175905549	0.174	chr8:7506377 [l]	0.003
chr11:67603353	1.011	chr6:93174331	0.174	chr13:19108457	0.003
chr1:156180191	1.008	chr8:90685000	0.171	chr3:149637482	0.003
chr16:5754387	1.008	chr8:47176032	0.168	chrY:6150210	0.003
chr12:58327458 [d]	0.999	chr5:44730486	0.146	chr19:21658430 [l]	0.003
chr17:17610927	0.996	chr3:53977513	0.143	chr4:152403433	0.003
chr2:207037794	0.990	chr4:156304761	0.130	chrX:55997734	0.003
chr2:30613471	0.990	chr3:125891263 [h]	0.124	chr3:195927338	0.003
chr11:60682422 [e]	0.990	chr19:20156337	0.115	chr10:22596165	0.003
chr5:30495147 [c]	0.987	chr6:169346284	0.115	chr12:8463351	0.003
chr5:30486652 [c]	0.959	chr19:27646453 [k]	0.081	chr2:228363318	0.003
chr18:4917278	0.959	chr3:113032475 [c]	0.065	chr6:151455209	0.003
chr4:9601620 [e]	0.946	chr9:63681608	0.056	chr7:101149594	0.003
chr7:16198690	0.946	chr16:32106927	0.053	chr17:80551141	0.003
chr15:101859976	0.934	chr16:8178760	0.050	chr5:43581390	0.003
chr12:29932053	0.931	chr7:104752822 [b]	0.043	chr12:86489826	0.003
chr9:108595573	0.921	chr11:61655012	0.034	chr5:1595901	0.003
chr11:10391308	0.915	chr14:55025504	0.031	chr1:156180355	0.003
chr1:89066862	0.912	chr6:111255096	0.025	chr12:6885661	0.003
chr17:5074679	0.912	chr5:1595807	0.025	chr7:83994076	0.003

chr10:68526637	0.903	chr8:139462993 [b]	0.025	chr5:18579423	0.003
chr5:55572040	0.897	chr1:10427662	0.022	chr18:26201669	0.003
chr1:52007212	0.881	chr7:16197472	0.022	chr11:118721014 [d]	0.003
chr6:52923641	0.881	chr7:23040824	0.022	chr3:129754939	0.003
chr8:37193367	0.872	chr5:116822099	0.022	chr20:34670264	0.003
chr10:99256369 [m]	0.872	chr4:62941846	0.019	chr2:228363641	0.003
chr19:27637589 [k]	0.866	chr16:23600524	0.019	chr1:10427810	0.003
chr9:69763136	0.859	chr1:75378054	0.019	chr6:143332028	0.003
chr19:44595064	0.859	chr3:195927426	0.019	chr19:23693587	0.003
chr16:23600444	0.856	chr7:46266002 [c]	0.012	chr19:29364879 [e]	0.003
chr15:65226541	0.850	chr1:209142278	0.009	chr3:75752749	0.003
chr2:230843638	0.847	chr9:40909063	0.009	chr6:78860274	0.003
chr4:119342533	0.841	chr5:156657624 [f]	0.009	chr1:156180513	0.003
chr8:143832958	0.838	chr13:19601303	0.009	chr10:99827959 [o]	0.003
chrX:145720840	0.813	chr8:144796862	0.009	chr7:50900109	0.003
chr11:24447053	0.813	chr7:101149701	0.009	chr8:131772168	0.003
chr6:134659109	0.794	chr10:42120525	0.009	chr10:65409229	0.003
chr6:78859756	0.794	chr20:35262123	0.009	chr16:20180721	0.003
chr12:55334400 [n]	0.785	chr8:143849708	0.009	chr11:63527970	0.003
chr11:67867963	0.769	chr19:37629876	0.006	chr3:65743670	0.003
chr6:33809959	0.757	chr12:56400360	0.006		

[a] Based on the NGS analysis, the clone number in each HERV-K integration site is divided by the total number in all HERV-K integration sites.

[b] Consistent with a previous report from John M. Coffin's group (1).

[c] Consistent with a previous report from Jack Lenz's group (2).

[d] Consistent with a previous report from Javier Costas (3).

[e] Consistent with a previous report from Robert Belshaw's group (4).

[f] Consistent with a previous report from Masao Ono's group (5).

[g] Consistent with a previous report from John M. Coffin's group (6).

[h] Consistent with a previous report from Yoshihiro Jinno's group (7).

[i] Consistent with a previous report from Dixie L. Mager's group (8).

[j] Consistent with a previous report from Peter J. Park's group (9).

[k] Consistent with a previous report from Ralf R. Tonjes's group (10).

[l] Consistent with a previous report (11).

[m] Consistent with a previous report from John M. Coffin's group (12).

[n] Consistent with a previous report from Robert Belshaw's group (13).

[o] Consistent with a previous report from Catriona Macfarlane's group (14).

Table S2. Loci of new HERV-K integration sites in HeLa and HERV-K-transfected HeLa cells.

Locus	Coordinate GRCh38/hg38	Sequences	Number of hits (100%)[a]
1p36.21	chr1:15,112,068	CCTACAGTAAGGTTCACAGTACACTGAAGAGTTAAGATCAAGACTT<br>TGGAGTGTCAGAAGCCTGAGTTCAGATCTTGAT	1
2q13	chr2:111,999,744	CCTACAGTGTGAGCAGTAAAGCTTTTTAATCACCCGGGTGCAGGT<br>GGGCTGAGTCCGAAAAAGGAGTCAGCAAAGGATGGTGGAATTATC<br>ATTAGTTCTTACAGGTTTGGGATAGGCAGTGGGGTTA*CCAAAATC<br>CCAAATTCACCTGGGG	0
3p26.1	chr3:4,366,476	CCTACAATGGAAGATGAAATGAATGAAATGAAGCGAGAGGGGAAG<br>TTTAGAGAAAAAAGAATAAAAAGAAATGAGC	0
6p22.3	chr6:23,230,703	CCTACAGCGTGGTGGCGGGCGCCTGTAGTCCCAGCTATTCAGGA<br>GGCTGAGGCAGGAGAATCGCTTGAACCTGGGA	2
7p21.2	chr7:16,217,694	CCTACAATGGAAGATGAAATGAATGAAATGAAGCGAGAAGGGAAG<br>TTTAGAGAAAAAAGAATAAAAAGAAATGAGC	N.S.[b]
7p22.1	chr7:4,599,432	CCTACATCTGGTGCCCAACGTGGAGGCTTTTCTCTAGGGTGAAGG<br>TACGCTCGAGCGTGGTCATTGAGGACAAGTC	12
8p11.1	chr8:43,312,043	CCTACAATGGAAAATGAAATGAATGAAATGAAGCGAGAAGGGAAG<br>TTTAGAGAAAAAAGAATAAAAAGAAATGAGC	N.S.[b]
8q24.22	chr8:133,275,187	CCTACACCAAGATTTTGGAGACTGCTGAAAATCTGGGGGACGCTT<br>T	1
10p15.1	chr10:6,263,616	CCTACACTGGCCCCAGAATTACAATGCAGGACAAAGAAGTGTGGG<br>TGTGGAGCTGAGA	1
10q11.21	chr10:42,101,508	CCTACATGATAATTCCATTCGATTCTATGCGATGATTCCATTCCTTTC<br>CATTAGAAGATGATTCCATTCGAGACCA	2
11q13.4	chr11:71,800,891	CCTACACCAAGTCCCTAACTCCCTCACTTGGACTTGAGACCCTTC<br>ACAACCCAGCAGCGCTCCGCCTCCAACTTGA	1
11q22.1	chr11:101,696,031	CCTACATCTGGTGCCCAACGTGGAGGCTTTTCTCTAGGGTGAAGG<br>TACGCTCGAGCGTGGTCATTGAGGACAAGTC	12
12p13.31	chr12:8,231,534	CCTACACCAAGTCCCTAACTCCCTCACTTGGACTTGAGACCCTTC<br>ACAACCCAGCAGCGCTCCGCCTCCAACGTGA	1
12p13.33	chr12:768,683	CCTACACCTCATTAATGTTTTAAACATCTGCCATTCTTATTCTCTTAA<br>TGAGTAAAGTTAACTTCTTCAACAACCAGTACTTAGCTAAATAT	1
12q23.1	chr12:100,854,613	CCTACACTCTTTAGTTGGTGTCTTTTTTGTTCCTTTTCAAAGCAACC<br>TTTTATTCCCACAGAGTTATAAACTAGTCCTTTCTACTCATTCACTG	1
13q22.2	chr13:76,617,585	CCTACAATGGAAGATGAAATGAATGAAATGAAGCGAGAAGGGAAG<br>TTTAGAGAAAAAAGAATAAAAGGAAATGAGCAAAGCCTCCAAGAAA<br>TATGGGCCTATGTGAAAAGACCAAATCAACGTCAGATTGGTGAACC<br>GGAAAGGCAGGGGG	0
16p11.2	chr16:30,785,310	CCTACAGCCTGGGGAAACCTCTCCAAGGTCAAGGCTGGGAAGTG<br>GCAGAGCTAAGATGATAAAAGCAGGGCT	1
16p12.3	chr16:20,180,721	CCTACATGGCACAATCTCAGCTCACTGCAACCTCTGCCTCCCAAG<br>TTCAAGCGATTATCCTGCCTCAGCCTCCTGAAAAGCTAGCATTACA	0
16p13.3	chr16:1,748,182	CCTACAACGGCTGCCCTCGGAACCTGAGTCTGGTGGGCTGGAGC<br>TGGACCACCTGCTAGGTGGGGATGATCAGAAA	1
16q11.2	chr16:46,398,910	CCTACACTCCATTCGAGTCCATTCGATGATTCCACTCGATTCCATTA<br>GATGATTCCATTGGAGTCCATTTGATTGT	1
17p13.1	chr17:8,072,100	CCTACACCCACCAGCAACCTAGTGGCCACAAGCCCTGCCACCTA<br>GGAGCCCAGCCTGCTAAGCTAAGTGCATCCTC	1
18p11.21	chr18:15,163,428	CCTACAAAGAAGTGTGAAAAAAGAAATATCTGTTGTTTTTTAGTCAA<br>CCGGTTTATGTTATTTGGTTATAAGAGTC	1
19p12	chr19:21,658,739	CCTACACTCTATAATTTTCTTACACCTAAGGTTTATCTTTAGACTAAC<br>ATATATTGAACTCTATGTAAATCAAAAC	1
20q13.2	chr20:51,642,183	CCTACACTCACTCTGTCACCTAGGCTGGAGTGCAGTGGTGCGATC<br>TCGGCTCATTGCAACCTCCACCTCCCGGGTT	1
Xp11.23	chrX:49,126,351	CCTACAGGCAGGCGCCTGTGGTTCCAGCTACTCAGGAGGCTGAG<br>GCATGAGAGATCGGAAGAGCGGTTCAGCCGGAATGCCGAGACCG<br>TCCTGAGCACCCCGATTCCGTCCTTCTGTTTGCAAAAAAAAAATTA<br>TACTTCCATATACCTTA	0
Xq13.3	chrX:75,233,189	CCTACAATGGAAGATGAAATGAATGAAATGAAGCGAGAAGGGAAG<br>TTTAGAGAAAAAAGAATAAAAAGAAATGAGC	N.S.[b]
Xq24	chrX:120,549,077	CCTACACCAAGTGGTAATGACTTAAATGTAACACCAGAATTCCTTTC<br>TCACCACAACAGAAGAAAAAAATTCCATTAAAATGAACTC	1

[a] Number of 100% identity and query cover using basic local alignment search tool within the NIH website.

[b] "No significant similarity found" that indicate short query sequences or filtering using basic local alignment search tool within the NIH website are as shown in N.S..

The sequences of 3'-U5 are shown as purple letters.

The mismatch nucleotides with database are shown as red letters.

Table S3. Sequences of new HERV-K integration sites in HERV-K-transfected HeLa cells.

Locus	Coordinate GRCh38/hg38	Sequences
**Universal[a], HeLa, HeLa-inBLC**		
11q13.4	chr11:71,800,891	ACCCACCCCTACACCAAGTCCCTAACTCCCTCACTTGGACTTGAGACCCTTCACAACCCAGCAGCGCTCCGCCTCCAACTTGACATCATGCTTTCTGGAAA
12p13.31	chr12:8,231,534	TTTCCAGAAAGCATGATGTCACGTTGGAGGCGGAGCGCTGCTGGGTTGTGAAGGGTCTCAAGTCCAAGTGAGGGAGTTAGGGACTTGGTGTAGGGGTGGGT
12q23.1	chr12:100,854,613	N.D.
17p13.1	chr17:8,072,100	N.D.
18p11.21	chr18:15,163,428	ACCCACCCCTACAAAGAAGTGTGAAAAAAGAAATATCTGTTGTTTTTTAGTCAACCGGTTTATGTTATTTGGTTATAAGAGTCCAAATAGACCAAGATATT
19p12[c]	chr19:21,658,739	AACACATGCAGACGTTTAGTTTTGATTTACATAGAGTTCAATATATGTTAGTCTAAAGATAAACCTTAGGTGTAAGAAAATTATAGAGTGTAGGGGTGGGT
20q13.2	chr20:51,642,183	N.D.
**Specific[b], HeLa-inBLC**		
1p36.21	chr1:15,112,068	ACCCACCCCTACAGTAAGGTTCACAGTACACTGAAGAGTTAAGATCAAGACTTTGGAGTGTCAGAAGCCTGAGTTCAGATCTTGATTCTGCCGCTAACAGC
8q24.22	chr8:133,275,187	ACCCACCCCTACACCAAGATTTTGGAGACTGCTGAAAATCTGGGGGACGCTTTCATCAATCTCTTGAGTCCCTAACACCTTCCAATCTAAGAAGCCAAATC
10p15.1	chr10:6,263,616	ACCCACCCCTACACTGGCCCCAGAATTACAATGCAGGACAAAGAAGTGTGGGTGTGGAGCTGAGAGACAAGAGCTTAATAATGGTTATTTTCTTCTGTTTT
12p13.33	chr12:768,683	ACCCACCCCTACACCTCATTAATGTTTTAAACATCTGCCATTCTTATTCTCTTAATGAGTAAAGTTAACTTCTTCAACAACCAGTACTTAGCTAAATATTA
16p11.2	chr16:30,785,310	ACCCACCCCTACAGCCTGGGGAAACCTCTCCAAGGTCAAGGCTGGGAAGTGGCAGAGCTAAGATGATAAAAGCAGGGCTCCATATCTGGCTCACCCCTGTC
16p13.3	chr16:1,748,182	CAGCCTGGGAAGAGCTGTTTTCTGATCATCCCCACCTAGCAGGTGGTCCAGCTCCAGCCCACCAGACTCAGGTTCCGAGGGCAGCCGTTGTAGGGGTGGGT
16q11.2	chr16:46,398,910	CGAATGGAATCGAATGGAACAATCAAATGGACTCCAATGGAATCATCTAATGGAATCGAGTGGAATCATCGAATGGACTCGAATGGAGTGTAGGGGTGGGT
Xq24	chrX:120,549,077	ACCCACCCCTACACCAAGTGGTAATGACTTAAATGTAACACCAGAATTCCTTTCTCACCACAACAGAAGAAAAAAATTCCATTAAAATGAACTCTAGGGGG

[a] Universal HERV-K integration sites in HeLa cells.
[b] Different HERV-K integration sites between HeLa and HERV-K/inBLC-transfected HeLa cells.
[c] Consistent with a previous report (11).
The sequences of 3'-U5 are shown as purple letters.

Table S4. The sequence of HERV-K/BLC genome with integration sites at 8q24.22 in HERV-K-transfected HeLa cells.

Outline	Sequence
8q24.22<br>TSD<br>5'LTR<br>Gag<br>Pro<br>Pol<br>BLC<br>SV40<br>3'LTR<br>TSD<br>8q24.22	TCTGAAGGAAATTCTACAGCAACCCTACACCCAAGTGTGGGAAAAGCAAGAGAGATCAGATTGTTACTGTGTC<br>TGTGTAGAAAGAAGTAGACATAGGAGACTCCATTTTGTTATGTACTAAGAAAAATTCTTCTGCCTTGAGATTCTGT<br>TAATCTATGACCTTACCCCCAACCCCGTGCTCTCTGAAACGTGTGCTGTGTCAACTCAGAGTTGAATGGATTAAGG<br>GCGGTGCAGGATGTGCTTTGTTAAACAGATGCTTGAAGGCAGCATGCTCCTTAAGAGTCATCACCACTCCCTAATC<br>TCAAGTACCCAGGGACACAAAAACTGCGGAAGGCCGCAGGGACCTCTGCCTAGGAAAGCCAGGTATTGTCCAA<br>GGTTTCTCCCCATGTGATAGTCTGAAATATGGCCTCGTGGGAAGGGAAAGACCTGACCGTCCCCCAGCCCGACAC<br>CCGTAAAGGGTCTGTGCTGAGGAGGATTAGTAAAAGAGGAAGGAATGCCTCTTGCAGTTGAGACAAGAGGAAG<br>GCATCTGTCTCCTGCCTGTCCCTGGGCAATGGAATGTCTCGGTATAAAACCCGATTGTATGCTCCATCTACTGAGAT<br>AGGGAAAAACCGCCTTAGGGCTGGAGGTGGGACCTGCGGGCAGCAATACTGCTTTGTAAAGCACTGAGATGTT<br>TATGTGTATGCATATCTAAAAGCACAGCACTTAATCCTTTACATTGTCTATGATGCAAAGACCTTTGTTCACGTGTTT<br>GTCTGCTGACCCTCTCCCCACAATTGTCTTGTGACCCTGACACATCCCCCTCTTTGAGAAACACCCACAGATGATC<br>AATAAATACTAAGGGAACTCAGAGGCTGGCGGGATCCTCCATATGCTGAACGCTGGTTCCCCGGGTCCCCTTATTT<br>CTTTCTCTATACTTTGTCTCTGTGTCTTTTTCTTTTCCAAATCTCTCGTCCCACCTTACGAGAAACACCCACAGGTGT<br>GTAGGGGCAACCCACCCCTACATCTGGTGCCCAACGTGGAGGCTTTTCTCTAGGGTGAAGGTACGCTCGAGCGT<br>GGTCATTGAGGACAAGTCGACGAGAGATCCCGAGTACGTCTACAGTCAGCCTTACGGTAAGCTTGTGCGCTCGG<br>AAGAAGCTAGGGTGATAATGGGGCAAACTAAAAGTAAAATTAAAAGTAAATATGCCTCTTATCTCAGCTTTATTAA<br>AATTCTTTTAAAAAGAGGGGGAGTTAAAGTATCTACAAAAAATCTAATCAAGCTATTTCAAATAATAGAACAATTTT<br>GCCCATGGTTTCCAGAACAAGGAACTTTAGATCTAAAAGATTGGAAAAGAATTGGTAAGGAACTAAAACAAGCA<br>GGTAGGAAGGGTAATATCATTCCACTTACAGTATGGAATGATTGGGCCATTATTAAAGCAGCTTTAGAACCATTTC<br>AAACAGAAGAAGATAGCGTTTCAGTTTCTGATGCCCCTGGAAGCTGTATAATAGATTGTAATGAAAACACAAGGA<br>AAAAATCCCAGAAAGAAACGGAAGGTTTACATTGCGAATATGTAGCAGAGCCGGTAATGGCTCAGTCAACGCAA<br>AATGTTGACTATAATCAATTACAGGAGGTGATATATCCTGAAACGTTAAAATTAGAAGGAAAAGGTCCAGAATTAG<br>TGGGGCCATCAGAGTCTAAACCACGAGGCACAAGTCCTCTTCCAGCAGGTCAGGTGCCCGTAACATTACAACCTC<br>AAAAGCAGGTTAAAGAAAATAAGACCCAACCGCCAGTAGCCTATCAATACTGGCCTCCGGCTGAACTTCAGTATC<br>GGCCACCCCCAGAAAGTCAGTATGGATATCCAGGAATGCCCCCAGCACCACAGGGCAGGGCGCCATACCCTCAG<br>CCGCCCACTAGGAGACTTAATCCTACGGCACCACCTAGTAGACAGGGTAGTGAATTACATGAAATTATTGATAAAT<br>CAAGAAAGGAAGGAGATACTGAGGCATGGCAATTCCCAGTAACGTTAGAACCGATGCCACCTGGAGAAGGAGC<br>CCAAGAGGGAGAGCCTCCCACAGTTGAGGCCAGATACAAGTCTTTTTCGATAAAAATGCTAAAAGATATGAAAG<br>AGGGAGTAAAACAGTATGGACCCAACTCCCCTTATATGAGGACATTATTAGATTCCATTGCTCATGGACATAGACTC<br>ATTCCTTATGATTGGGAGATTCTGGCAAAATCGTCTCTCTCACCCTCTCAATTTTTACAATTTAAGACTTGGTGGATT<br>GATGGGGTACAAGAACAGGTCCGAAGAAATAGGGCTGCCAATCCTCCAGTTAACATAGATGCAGATCAACTATTA<br>GGAATAGGTCAAAATTGGAGTACTATTAGTCAACAAGCATTAATGCAAAATGAGGCCATTGAGCAAGTTAGAGCT<br>ATCTGCCTTAGAGCCTGGGAAAAAATCCAAGACCCAGGAAGTACCTGCCCCTCATTTAATACAGTAAGACAAGGT<br>TCAAAAGAGCCCTATCCTGATTTTGTGGCAAGGCTCCAAGATGTTGCTCAAAAGTCAATTGCCGATGAAAAAGCC<br>CGTAAGGTCATAGTGGAGTTGATGGCATATGAAAACGCCAATCCTGAGTGTCAATCAGCCATTAAGCCATTAAAAG<br>GAAAGGTTCCTGCAGGATCAGATGTAATCTCAGAATATGTAAAAGCCTGTGATGGAATCGGAGGAGCTATGCATA<br>AAGCTATGCTTATGGCTCAAGCAATAACAGGAGTTGTTTTAGGAGGACAAGTTAGAACATTTGGAGGAAAATGTT<br>ATAATTGTGGTCAAATTGGTCACTTAAAAAAGAATTGCCCAGTCTTAAACAAACAGAATATAACTATTCAAGCAAC<br>TACAACAGGTAGAGAGCCACCTGACTTATGTCCAAGATGTAAAAAAGGAAAACATTGGGCTAGTCAATGTCGTTC<br>TAAATTTGATAAAAATGGGCAACCATTGTCGGGAAACGAGCAAAGGGGCCAGCCTCAGGCCCCACAACAAACTG<br>GGGCATTCCCAATTCAGCCATTTGTTCCTCAGGGTTTTCAGGGACAACAACCCCCACTGTCCCAAGTGTTTCAGG<br>GAATAAGCCAGTTACCACAATACAACAATTGTCCCCCGCCACAAGCGGCAGTGCAGCAGTAGATTTATGTACTATA<br>CAAGCAGTCTCTCTGCTTCCAGGGGAGCCCCACAAAAAATCCCCACAGGGGTATATGGCCCCCTGCCTGAGGG<br>GACTGTAGGACTAATCTTGGGAAGATCAAGTCTAAATCTAAAAGGAGTTCAAATTCATACTAGTGTGGTTGATTCA<br>GACTATAAAGGCGAAATTCAATTGGTTATTAGCTCTTCAATTCCTTGGAGTGCCAGTCCAGGAGACAGGATTGCTC<br>AATTATTACTCCTGCCATATATTAAGGGTGGAAATAGTGAAATAAAAAGAATAGGAGGGCTTGGAAGCACTGATCC<br>AACAGGAAAGGCTGCATATTGGGCAAGTCAGGTCTCAGAGAACAGACCTGTGTGTAAGGCCATTATTCAAGGAA<br>AACAGTTTGAAGGGTTGGTAGACACTGGAGCAGATGTCTCTATCATTGCTTTAAATCAGTGGCCAAAAAATTGGC<br>CTAAACAAAAGGCTGTTACAGGACTTGTCGGCATAGGCACAGCCTCAGAAGTGTATCAAAGTACGGAGATTTTAC<br>ATTGCTTAGGGCCAGATAATCAAGAAAGTACTGTTCAGCCAATGATTACTTCAATTCCTCTTAATCTGTGGGGTCG<br>AGATTTATTACAACAATGGGGTGCGGAAATCACCATGCCCGCTCCATTATATAGCCCCACGAGTCAAAAAATCATG<br>ACCAAGATGGGATATATACCAGGAAAGGGACTAGGGAAAAATGAAGATGGCATTAAAGTTCCAGTTGAGGCTAA<br>AATAAATCAAGAAAGAGAAGGAATAGGGTATCCTTTTTAGGGGCGGCCACTGTAGAGCCTCCTAAACCCATACCA<br>TTAACTTGGAAAACAGAAAAACCGGTGTGGGTAAATCAGTGGCCGCTACCAAAACAAAAACTGGAGGCTTTACA<br>TTTATTAGCAAATGAACAGTTAGAAAAGGGTCATATTGAGCCTTCGTTCTCACCTTGGAATTCTCCTGTGTTTGTAA<br>TTCAGAAGAAATCAGGCAAATGGCGTATGTTAACTGACTTAAGGGCTGTAAACGCCGTAATTCAACCCATGGGGC<br>CTCTCCAACCCGGGTTGCCCTCTCCGGCCATGATCCCAAAAGATTGGCCTTTAATTATAATTGATCTAAAGGATTGC<br>TTTTTTACCATCCCTCTGGCAGAGCAGGATTGCGAAAAATTTGCCTTTACTATACCAGCCATAAATAATAAAGAACC<br>AGCCACCAGGTTTCAGTGGAAAGTGTTACCTCAGGGAATGCTTAATAGTCCAACTATTTGTCAGACTTTTGTAGGT<br>CGAGCTCTTCAACCAGTTAGAGAAAAGTTTTCAGACTGTTATATTATTCATTATATTGATGATATTTTATGTGCTGCA

	GAAACGAAAGATAAATTAATTGACTGTTATACATTTCTGCAAGCAGAGGTTGCCAATGCTGGACTGGCAATAGCAT<br>CTGATAAGATCCAAACCTCTACTCCTTTTCATTATTTAGGGATGCAGATAGAAAATAGAAAAATTAAGCCACAAAA<br>AATAGAAATAAGAAAAGACACATTAAAAACACTAAATGATTTTCAAAAATTACTAGGAGATATTAATTGGATTCGG<br>CCAACTCTAGGCATTCCTACTTATGCCATGTCAAATTTGTTCTCTATCTTAAGAGGAGACTCAGACTTAAATAGTAA<br>AAGAATGTTAACCCCAGAGGCAACAAAAGAAATTAAATTAGTGGAAGAAAAAATTCAGTCAGCGCAAATAAATA<br>GAATAGATCCCTTAGCCCCACTCCAACTTTTGATTTTTGCCACTGCACATTCTCCAACAGGCATCATTATTCAAAATA<br>CTGATCTTGTGGAGTGGTCATTCCTTCCTCACAGTACAGTTAAGACTTTTACATTGTACTTGGATCAAATAGCTACA<br>TTAATCGGTCAGACAAGATTACGAATAATAAAATTATGTGGAAATGACCCAGACAAAATAGTTGTCCCTTTAACCA<br>AGGAACAAGTTAGACAAGCCTTTATCAATTCTGGTGCATGGCAGATTGGTCTTGCTAATTTTGTGGGAATTATTGA<br>TAATCATTACCCAAAAACAAAGATCTTCCAGTTCTTAAAATTGACTACTTGGATTCTACCTAAAATTACCAGACGTG<br>AACCTTTAGAAAATGCTCTAACAGTATTTACTGATGGTTCCAGCAATGGAAAAGCAGCTTACACAGGGCCGAAAG<br>AACGAGTAATCAAAACTCCATATCAATCGGCTCAAAGAGCAGAGTTGGTTGCAGTCATTACAGTGTTACAAGATTT<br>TGACCAACCTATCAATATTATATCAGATTCTGCATATGTAGTACAGGCTACAAGGGATGTTGAGACAGCTCTAATTA<br>AATATAGCATGGATGATCAGTTAAACCAGCTATTCAATTTATTACAACAAACTGTAAGAAAAAGAAATTTCCCATTT<br>TATATTACTCATATTCGAGCACACACTAATTTACCAGGGCCTTTGACTAAAGCAAATGAACAAGCTGACTTACTGGT<br>ATCATCTGCACTCATAAAAGCACAAGAACTTCATGCTTTGACTCATGTAAATGCAGCAGGATTAAAAAACAAATTT<br>GATGTCACATGGAAACAGGCAAAAGATATTGTACAACATTGCACCCAGTGTCAAGTCTTACACCTGCCCACTCAA<br>GAGGCAGGAGTTAATCCCAGAGGTCTGTGTCCTAATGCATTATGGCAAATGGATGTCACGCATGTACCTTCATTTG<br>GAAGATTATCATATGTTCATGTAACAGTTGATACTTATTCACATTTCATATGGGCAACTTGCCAAACAGGAGAAAGT<br>ACTTCCCATGTTAAAAAACATTTATTGTCTTGTTTTGCTGTAATGGGAGTTCCAGAAAAAATCAAAACTGACAATG<br>GACCAGGATATTGTAGTAAAGCTTTCCAAAAATTCTTAAGTCAGTGGAAAATTTCACATACAACAGGAATTCCTTA<br>TAATTCCCAAGGACAGGCCATAGTTGAAAGAACTAATAGAACACTCAAAACTCAATTAGTTAAACAAAAAGAAGG<br>GGGAGACAGTAAGGAGTGTACCACTCCTCAGATGCAACTTAATCTAGCACTCTATACTTTAAATTTTTTAAACATTT<br>ATAGAAATCAGACTACTACTTCTGCAGAACAACATCTTACTGGTAAAAAGAACAGCCCACATGAAGGAAAACTAA<br>TTTGGTGGAAAGATAATAAAAATAAGACATGGGAAATAGGGAAGGTGATAACGTGGGGGAGAGGTTTTGCTTGT<br>GTTTCACCAGGAGAAAATCAGCTTCCTGTTTGGATACCCACTAGACATTTGAAGTTCTACAATGAACCCATCGGAG<br>ATGCAAAGAAAAGCACCTCCGCGGAGACGGAGACACCGCAATCGAGCACCGTTGACTCACAAGATGAACAAAA<br>TGGTGACGTCAGAAGAACAGATGAAGTTGCCATCCACCAAGAAGGCAGAGCCGCCGACTTGGGCACAACTAAA<br>GAAGCTGACGCAGTTAGCTACAAAATATCTAGAGAACACAAAGGTGACACAAACCCCAGAGAGTATGCTGCTTG<br>CAGCCTTGATGATTGTATCAATGGTGGTAAGTCTCCCTATGCCTGCAGGAGCAGCTGCAGCTAACTATACCTACTG<br>GGCCTATGTGCCTTTCCCGCCCTTAATTCGGGCAGTCACATGGATGGATAATCCTATAGAAGTATATGTTAATGATA<br>GTGTATGGATGCGGCCGCAAATCATGCTGCTATAAAGACACATGCACACGTATGTTTATTGCGGCACTATTCACAAT<br>AGCAAAGACTTGGAACCAACCCAAATGTCCAACAATGATAGACTGGATTAAGAAAATGTGGCACATATACACCAT<br>GGAATACTATGCAGCCATAAAAAATGATGAGTTCATATCCTTTGTAGGGACATGGATGAAATTGGAAACCATCATT<br>CTCAGTAAACTATCGCAAGAACAAAAACCAAACACCGCATATTCTCACTCATAGGTGGGAATTGAACAATGAGA<br>TCACATGGACACAGGAAGGGGAATATCACACTCTGGGGACTGTGGTGGGGTCGGGGGAGGGGGGAGGGATAG<br>CATTGGGAGATATACCTAATGCTAGATGACACATTAGTGGGTGCTGCGCACCAGCATGGCACATGTAATCCCTGCA<br>GGCATAAGCGATCCGAACCCAACGACCCAACACCCGTGCGTTTTATTCTGTCTTTTTATTGCCGATCCCCGGTCAC<br>CCGCCCCGACTCTAGAATTATTAGCCCTCCCACACATAACCAGAGGGCAGCAATTCACGAATCCCAACTGCCGTCG<br>GCTGTCCATCACTGTCCTTCACTATGGCTTTGATCCCAGGATGCAGATCGAGAAGCACCTGTCGGCACCGTCCGCA<br>GGGGCTCAGGATGCCCCTGTTCTCATTTCCGATCGCGACGATACAAGTCAGGTTGCCAGCTGCCGCAGCAGCAG<br>CAGTGCCCAGCACCACGAGTTCTGCACAAGGTCCCCCAGTAAAATGATATACATTGACACCAGTGAAGATGCGGC<br>CGTCGCTAGAGAGAGCTGCGCTGGCGACGCTGTAGTCTTCAGAGATGGGGATGCTGTTGATTGTAGCCGTTGCT<br>CTTTCAATGAGGGTGGATTCTTCTTGAGACAAAGGCTTGGCCATGGTGGCTTTACCAACAGTACCGGATTGCCAA<br>GCTTTTTGCAAAAGCCTAGGCCTCCAAAAAAGCCTCCTCACTACTTCTGGAATAGCTCAGAGGCCGAGGCGGCC<br>TCGGCCTCTGCATAAATAAAAAAAATTAGTCAGCCATGGGGCGGAGAATGGGCGGAACTGGGCGGAGTTAGGG<br>GCGGGATGGGCGGAGTTAGGGGCGGGACTATGGTTGCTGACTAATTGAGATGCATGCTTTGCATACTTCTGCCTG<br>CTGGGGAGCCTGGGGACTTTCCACACCTGGTTGCTGACTAATTGAGATGCATGCTTTGCATACTTCTGCCTGCTG<br>GGGAGCCTGGGGACTTTCCACACCCTAACTGACACACATTCCACAGCTGGTTCTTTCCGCCTCAGAAGGTACCTA<br>ACCAAGTTCCTCTTTCAGAGGTTATTTCAGGCCATGGTGCTGCGCAGGCGGCCGCATCTGGCCCCATAGATGATC<br>GCTGCCCTGCCAAACCTGAGGAAGAAGGGATGATGATAAATATTTCCATTGGGTATCGTTATCCTCCTATTTGCCTA<br>GGGAGAGCACCAGGATGTTTAATGCCTGCAGTCCAAAATTGGTTGGTAGAAGTACCTACTGTCAGTCCCATCAGT<br>AGATTCACTTATCACATGGTAAGCGGGATGTCACTCAGGCCACGGGTAAATTATTTACAAGACTTTTCTTATCAAA<br>GATCATTAAAATTTAGACCTAAAGGGAAACCTTGCCCCAAGGAAATTCCCAAAGAATCAAAAAATACAGAAGTTT<br>TAGTTTGGGAAGAATGTGTGGCCAATAGTGCGGTGATATTACAAAACAATGAATTTGGAACTATTATAGATTGGGC<br>ACCTCGAGGTCAATTCTACCACAATTGCTCAGGACAAACTCAGTCGTGTCCAAGTGCACAAGTGAGTCCAGCTGT<br>TGATAGCGACTTAACAGAAAGTTTAGACAAACATAAGCATAAAAAATTGCAGTCTTTCTACCCTTGGGAATGGGG<br>AGAAAAAGGAATCTCTACCCCAAGACCAAAAATAGTAAGTCCTGTTTCTGGTCCTGAACATCCAGAATTATGGAG<br>GCTTACTGTGGCCTCACACCACATTAGAATTTGGTCTGGAAATCAAACTTTAGAAACAAGAGATCGTAAGCCATTT<br>TATACTGTCGACCTAAATTCCAGTCTAACAGTTCCTTTACAAAGTTGCGTAAAGCCCCCTTATATGCTAGTTGTAGG<br>AAATATAGTTATTAAACCAGACTCCCAGACTATAACCTGTGAAAATTGTAGATTGCTTACTTGCATTGATTCAACTTT<br>TAATTGGCAACACCGTATTCTGCTGGTGAGAGCAAGAGAGGGCGTGTGGATCCCTGTGTCCATGGACCGACCGT<br>GGGAGGCCTCACCATCCGTCCATATTTTGACTGAAGTATTAAAAGGTGTTTTAAATAGATCCAAAAGATTCATTTTT

	ACTTTAATTGCAGTGATTATGGGATTAATTGCAGTCACAGCTACGGCTGCTGTAGCAGGAGTTGCATTGCACTCTT<br>CTGTTCAGTCAGTAAACTTTGTTAATGATTGGCAAAAAAATTCTACAAGATTGTGGAATTCACAATCTAGTATTGAT<br>CAAAAATTGGCAAATCAAATTAATGATCTTAGACAAACTGTCATTTGGATGGGAGACAGACTCATGAGCTTAGAA<br>CATCGTTTCCAGTTACAATGTGACTGGAATACGTCAGATTTTTGTATTACACCCCAAATTTATAATGAGTCTGAGCAT<br>CACTGGGACATGGTTAGACGCCATCTACAGGGAAGAGAAGATAATCTCACTTTAGACATTTCCAAATTAAAAGAA<br>CAAATTTTCGAAGCATCAAAAGCCCATTTAAATTTGGTGCCAGGAACTGAGGCAATTGCAGGAGTTGCTGATGGC<br>CTCGCAAATCTTAACCCTGTCACTTGGGTTAAGACCATTGGAAGTACTACGATTATAAATCTCATATTAATCCTTGTG<br>TGCCTGTTTTGTCTGTTGTTAGTCTGCAGGTGTACCCAACAGCTCCGAAGAGACAGCGACCATCGAGAACGGGC<br>CATGATGACGATGGCGGTTTTGTCGAAAAGAAAAGGGGGAAATGTGGGGAAAAGCAAGAGAGATCAGATTGTT<br>ACTGTGTCTGTGTAGAAAGAAGTAGACATAGGAGACTCCATTTTGTTATGTACTAAGAAAAATTCTTCTGCCTTGA<br>GATTCTGTTAATCTATGACCTTACCCCCAACCCCGTGCTCTCTGAAACGTGTGCTGTGTCAACTCAGAGTTGAATG<br>GATTAAGGGCGGTGCAGGATGTGCTTTGTTAAACAGATGCTTGAAGGCAGCATGCTCCTTAAGAGTCATCACCAC<br>TCCCTAATCTCAAGTACCCAGGGACACAAAAACTGCGGAAGGCCGCAGGGACCTCTGCCTAGGAAAGCCAGGTA<br>TTGTCCAAGGTTTCTCCCCATGTGATAGTCTGAAATATGGCCTCGTGGGAAGGGAAAGACCTGACCGTCCCCCAG<br>CCCGACACCCGTAAAGGGTCTGTGCTGAGGAGGATTAGTAAAAGAGGAAGGAATGCCTCTTGCAGTTGAGACA<br>AGAGGAAGGCATCTGTCTCCTGCCTGTCCCTGGGCAATGGAATGTCTCGGTATAAAACCCGATTGTATGCTCCATC<br>TACTGAGATAGGGAAAAACCGCCTTAGGGCTGGAGGTGGGACCTGCGGGCAGCAATACTGCTTTGTAAAGCACT<br>GAGATGTTTATGTGTATGCATATCTAAAAGCACAGCACTTAATCCTTTACATTGTCTATGATGCAAAGACCTTTGTTC<br>ACGTGTTTGTCTGCTGACCCTCTCCCCACAATTGTCTTGTGACCCTGACACATCCCCCTCTTTGAGAAACACCCAC<br>AGATGATCAATAAATACTAAGGGAACTCAGAGGCTGGCGGGATCCTCCATATGCTGAACGCTGGTTCCCCGGGTC<br>CCCTTATTTCTTTCTCTATACTTTGTCTCTGTGTCTTTTTCTTTTCCAAATCTCTCGTCCCACCTTACGAGAAACACCC<br>ACAGGTGTGTAGGGGCAACCCACCCCTACACCAAGATTTTGGAGACTGCTGAAAAT

Table S5. Loci of new HERV-K integration sites in fibroblast and iPS cells.

Locus	Coordinate GRCh38/hg38	Sequences	Number of hits (100%)[a]
1p13.2	chr1:111,609,710	CCTACAACTGCAACCTCCGCCTCCT*AGTTCAAGTGATTCTCCTGC<br>CTCAGCCTCCTGAGTAGCTGGGATTACAGGC	0
1p13.2	chr1:113,755,874	CCTTCAGTTAGCCAGGATGGTCTCGATCTCCTGACCTCGTGATCC<br>ACCTGCCTCAGCCTCCCAAAGTGCTGGGAT	9
1p34.3	chr1:38,842,086	CCTTCAGTTAGCCAGGATGGTCTCGATCTCCTGACCTCGTGATCC<br>ACCTGCCTCAGCCTCCTGAGTAGCTGGGATT	0
1p35.1	chr1:32,614,846	CCTACAACTGCAACCTCCGCCTCCTGGGTTTAAGTGATTCCCCTG<br>CCTCAGCCTCC	1
2q22.3	chr2:147,700,152	CCTTCATACTACTTTTTTCTTCTGAAGTTTTTTTTGGGGGGTGGAAT<br>GGAGAAAGGAAGGAGAGGAAGGAGGTTGA	1
3q11.2	chr3:97,642,693	CCTACAATGGAAGATGAAATGAATGAAATGAAGCGAGAAGGGAAG<br>TTTAGAGAAAAAAGAATAAAAAGAAATGAGC	N.S.[b]
3q13.2	chr3:113,032,475	CCTTCATCTGGTGCCCAACGTGGAGGCTTTTCTCTAGGGTGAAGG<br>TACGCTCGAGCGTGGTCATTGAGGACAAGTC	12
4q22.1	chr4:91,979,478	CCTACAATGGAAGATGAAATGAATGAAATGAAGCGAGAAGGGAAG<br>TTTAGAGAAAAAAGAATAAAAAGAAATGAGC	N.S.[b]
4q25	chr4:111,308,304	CCTTCAGTTAGCCAGGATGGTCTCGATCTCCTGACCTCGTGATCC<br>ACCTGCCTCGGCTTCCCAAAGTGCTGGGATT	5
5p13.3	chr5:30,822,734	CCTACAATGGAAGATGAAATGAGTGAAATGAAGCGAGAAGGGAAG<br>TTTAGAGAAAAAAGAATAAAAAGAAATGAGA	N.S.[b]
5p13.3	chr5:32,212,988	CCTACATGGCACAATCTCAGCTCACTGCAACCTCTGCCTCCTGGG<br>TTCAAGCGATTCT*CTGCCTCAACCTCCCGAG	0
6q14.1	chr6:77,725,406	CCTACATCTGGTGCCCAACGTGGAGGCTTTTCTTTAGGGTGAAGG<br>TACGCTCGAGCGTGGTCATTGAGGACAAGTC	0
7p14.3	chr7:334,071,494	CCTACAATGGAAGATGAAATGAACGAAATGAAGCGAGAAGGGAAG<br>TTTAGAGAAAAAAGAAGAAAAAGAAATGAGC	N.S.[b]
8q13.1	chr8:66,953,856	CCTACAATGGAAGATGAAATGAATGAAATGAAGCGAGAAGGGAAG<br>TTTAGAGAAAAAAGAATAAAAAGAACTGAGC	N.S.[b]
9q21.13	chr9:73,987,206	CCTACAATGCACAAAAGGGGGAAAGATGTCTAATCTTTTTATTTTCA<br>ATTGGAAAATATGAAAATTTTCCATCAAA	1
9q22.33	chr9:99,500,005	CCTACAATGGAAGATGAAATGAATGAAATGAAGCGAGAAGGGAAG<br>TTTAGAGAAAAAAGAATAAAAAGAAATGAGC	N.S.[b]
10q24.2	chr10:99,256,369	CCTTCACATCTCATGGCAGAAGGGCAAAGAGAGGGCAAGAAAGA<br>GCAAGAGATCAAACTTGCAGGCTCAAGCCTTT	1
11q13.2	chr11:67,868,810	CCTACAGTGACCTTACCCCCAACCCCGTGCTCTCTGAAACATGTG<br>CTGTGTCAACTCAGAGTTAAATGGATTAAGG	12
11q13.3	chr11:70,471,356	CCTACATCCTCCAAATGGGGGAGAATGTGCTGGAAGCCTGACTGT<br>GTGTTTTGCGGCCCATGTGCATCTGGTGACC	1
12q12	chr12:43,919,854	CCTACAGTGGTTTTGATTTTCATGTTCTTTCTTTTGGTGACTTTATT<br>CTCAAGAATTGACTGATCAGCTCTAAATT	1
13q31.3	chr13:90,090,934	CCTACAAGTAGTTATATTAATAATAAAAATTATTTTTAAACTTAGAATT<br>TGATTCACATGATATTAAAGAGTCACA	1
15q11.2	chr15:23,715,336	CCTACAATGGAAGATGAAATGAATGAAATGAAGCGAGAAGGGAAG<br>TTTAGAGAAAAAAGAATAAAAAGAAATGAGC	N.S.[b]
15q14	chr15:36,348,199	CCTACATGACAAGACTGAACAACAGCTCTAGTAGTTTGCGAATTTT<br>AGATCCACTGTCATAATCTTGGTGGACATT	1
15q15.1	chr15:41,053,309	CCTTCAGTTAGCCAGGATGGTCTCGATTTCCTGACCTCATGATCCG<br>CCCACCTCAGCCTCCCAAAGTGCTGGGATT	3
16p12.3	chr16:18,135,887	CCTACAACTGCAACCTCCGCCTCC*TAGTTCAAGTGATTCTCCTGC<br>CTCAGCCTCCCAAGTAGCTGGGACCACAGGA	0
20q11.23	chr20:38,348,370	CCTACATGGCACAATCTCAGCTCACTGCAACCTCCGACTCCCTGG<br>TTCAAGTGATTCTCCTGCCTCAGCCTCCTGA	2
21p11.2	chr21:8,586,021	CCTACAAAGAAGTGTGAAAAAAGAAATATCTGTTGTTTTTTAGTCAA<br>CCGGTTTATGTTATTTGGTTATAAGAGTC	1
21q11.2	chr21:13,820,849	CCTACAGATTTCTAGCCTGAAAAATGGAGGAACAATGTCTGTCATA<br>TAAAGTTGCTGTAAATATAAAACCAGATAA	0
21q22.3	chr21:43,711,450	CCTTCATGCTCACGCCTGTAATCCCAGCACTTTGGGAGGCCGAGG<br>CGGGCGGATCACGAAGATCGGAAGAGCGGTT	0
Xq25	chrX:126,189,98	CCTACAATGGAAGATGAAATGAATGAAATGAAGCGAGAAGGGAAG<br>TTTAGAGAAAAAAGAATAAAAAGAAATGAGC	N.S.[b]

[a] Number of 100% identity and query cover using basic local alignment search tool within the NIH website.

[b] "No significant similarity found" that indicate short query sequences or filtering using basic local alignment search tool within the NIH website are as shown in N.S..

The sequences of 3'-U5 are shown as purple letters.

The mismatch nucleotides with database are shown as red letters.

The deletions of nucleotide are shown as *.

Table S6. Sequences of new HERV-K integration sites in iPS cells.

**Locus**	**Coordinate GRCh38/hg38**	**Sequences**
**Universal[a], fibroblast, iPS31, iPS41**		
1p35.1	chr1:32,614,846	CAGGCATGGTGGTTGGTGCCTGTAGTCCCACCTACTTGGGAGGCTGAGGCAGGGGAATCACTTAAACCCAGGAGGCGGAGGTTGCAGTTGTAGGGGTGGGT
10q24.2	chr10:99,256,369	GATTAATGCCATTTATAAAAAGGCTTGAGCCTGCAAGTTTGATCTCTTGCTCTTTCTTGCCCTCTCTTTGCCCTTCTGCCATGAGATGTGAAGGGGTGGGT
12q12[c]	chr12:43,919,854	N.D.
13q31.3[c]	chr13:90,090,934	ATACTTTAGATTCTTTAATGTGACTCTTTAATATCATGTGAATCAAATTCTAAGTTTAAAAATAATTTTTATTATTAATATAACTACTTGTAGGGGTGGGT
**Specific[b], iPS31, iPS41**		
2q22.3	chr2:147,700,152	ACCCAGAGTCTCAATATCTCAACCTCCTTCCTCTCCTTCCTTTCTCCATTCCACCCCCCAAAAAAAAACTTCAGAAGAAAAAAGTAGTATGAAGGGGTGGGT
11q13.3	chr11:70,471,356	ACCCACCCCTACATCCTCCAAATGGGGGAGAATGTGCTGGAAGCCTGACTGTGTGTTTTGCGGCCCATGTGCATCTGGTGACCTCTTTTGGAAATTCGAGG
15q14	chr15:36,348,199	TGCCCAAATCCTGATGACAATGTCCACCAAGATTATGACAGTGGATCTAAAATTCGCAAACTACTAGAGCTGTTGTTCAGTCTTGTCATGTAGGGGTGGGT

[a] Universal HERV-K integration sites in this donor.
[b] Different HERV-K integration sites between fibroblast and iPS cells
[c] Consistent with a previous report from Robert Belshaw's group (4).
The sequences of 3'-U5 are shown as purple letters.
